# The impact of death and dying on the personhood of medical students: a systematic scoping review

**DOI:** 10.1186/s12909-020-02411-y

**Published:** 2020-12-28

**Authors:** Chong Yao Ho, Cheryl Shumin Kow, Chin Howe Joshua Chia, Jia Ying Low, Yong Hao Melvin Lai, Sarah-Kei Lauw, Ashley Ern Hui How, Lorraine Hui En Tan, Xin Ling Lisa Ngiam, Natalie Pei Xin Chan, Tze Yin Joshua Kuek, Nur Haidah Ahmad Kamal, Jeng Long Chia, Ahmad Bin Hanifah Marican Abdurrahman, Min Chiam, Yun Ting Ong, Annelissa Mien Chew Chin, Ying Pin Toh, Stephen Mason, Lalit Kumar Radha Krishna

**Affiliations:** 1 Yong Loo Lin School of Medicine, National University of Singapore, 1E Kent Ridge Road, NUHS Tower Block, Level 11, Singapore, 119228 Singapore; 2 Division of Supportive and Palliative Care, National Cancer Centre Singapore, Level 4, 11 Hospital Crescent, Singapore, 169610 Singapore; 3 Lee Kong Chian School of Medicine, Nanyang Technological University, 59 Nanyang Dr, Experimental Medicine Building, Singapore, 636921 Singapore; 4 Division of Cancer Education, National Cancer Centre Singapore, Level 4, 11 Hospital Crescent, Singapore, 169610 Singapore; 5 Medical Library, National University of Singapore Libraries, Blk MD6, Centre, 14 Medical Dr, #05-01 for Translational Medicine, Singapore, 117599 Singapore; 6 Star PALS (Paediatric Advanced Life Support), HCA Hospice Care, Kwong Wai Shiu Hospital Singapore, 705 Serangoon Road, Block A #03-01, Singapore, 328127 Singapore; 7 Department of Family Medicine, Yong Loo Lin School of Medicine, National University of Singapore, 1E Kent Ridge Road, NUHS Tower Block, Level 11, Singapore, 119228 Singapore; 8 Palliative Care Institute Liverpool, Academic Palliative & End of Life Care Centre, University of Liverpool, Cancer Research Centre, University of Liverpool, 200 London Road, Liverpool, L3 9TA UK; 9 Duke-NUS Medical School, 8 College Road, Singapore, 169857 Singapore; 10 Centre of Biomedical Ethics, National University of Singapore, Blk MD11, 10 Medical Drive, #02-03, Singapore, 117597 Singapore; 11 PalC, The Palliative Care Centre for Excellence in Research and Education, PalC c/o Dover Park Hospice, 10 Jalan Tan Tock Seng, Singapore, 308436 Singapore

**Keywords:** Medical student, Dying patients, Personhood, Ring theory of personhood, Resilience, Organisational ethics, Medical schools, Undergraduate medical education

## Abstract

**Background:**

The re-introduction of medical students into healthcare systems struggling with the COVID-19 pandemic raises concerns as to whether they will be supported when confronted with death and dying patients in resource-limited settings and with reduced support from senior clinicians. Better understanding of how medical students respond to death and dying will inform educationalists and clinicians on how to best support them.

**Methods:**

We adopt Krishna’s Systematic Evidence Based Approach to carry out a Systematic Scoping Review (SSR in SEBA) on the impact of death and dying on medical students. This structured search process and concurrent use of thematic and directed content analysis of data from six databases (Split Approach) enhances the transparency and reproducibility of this review.

**Results:**

Seven thousand six hundred nineteen were identified, 149 articles reviewed and 52 articles included. The Split Approach revealed similar themes and categories that correspond to the Innate, Individual, Relational and Societal domains in the Ring Theory of Personhood.

**Conclusion:**

Facing death and dying amongst their patients affect how medical students envisage their personhood. This underlines the need for timely, holistic and longitudinal support systems to ensure that problems faced are addressed early. To do so, there must be effective training and a structured support mechanism.

## Background

With nearly 20 million reported cases worldwide and at least 730,000 deaths [1–4], the COVID-19 global pandemic has stressed healthcare systems and impacted medical education curricula in numerous countries [5]. It is against this backdrop that medical students in certain countries are being asked to step into clinical wards and bolster primary medical teams, in some cases with minimal supervision [6–10]. For many students, this uncertain environment will likely bring with it their first exposure to death and dying. Whilst many medical schools have incorporated palliative care into their formal curricula, a prevailing culture that sees death as a medical failure still remains [11–18]. Medical educators, too, continue to struggle with sufficiently preparing their students emotionally and mentally for the caring of their dying patients and families [19–22].

In light of this pandemic, this may be exacerbated as some medical students enter a system facing “death at unprecedented rates” [23]. As senior clinicians scramble to meet clinical demands, their ability to provide support and guidance to these students are likely to fall short [24]. Ill-equipped, these medical students may be forced to witness the acute distress of multiple patients dying in isolation and watch as families grapple with physical separation from their fading loved ones [25]. Better understanding of how medical students respond to death and dying will thus inform educationalists and clinicians on how to better support them during this pandemic and beyond.

### The need for this paper

A systematic scoping review (SSR) is proposed to map available data to guide the design of much needed support systems for these medical students [18, 26].

## Methods

An SSR allows for a structured approach to systematic extraction, synthesis of actionable and applicable information and a summary of available literature across a wide range of settings [27–30]. To overcome concerns about the transparency and reproducibility of SSRs, we adopt Krishna’s Systematic Evidence Based Approach (SEBA) [31–37].

**Fig. 1:**
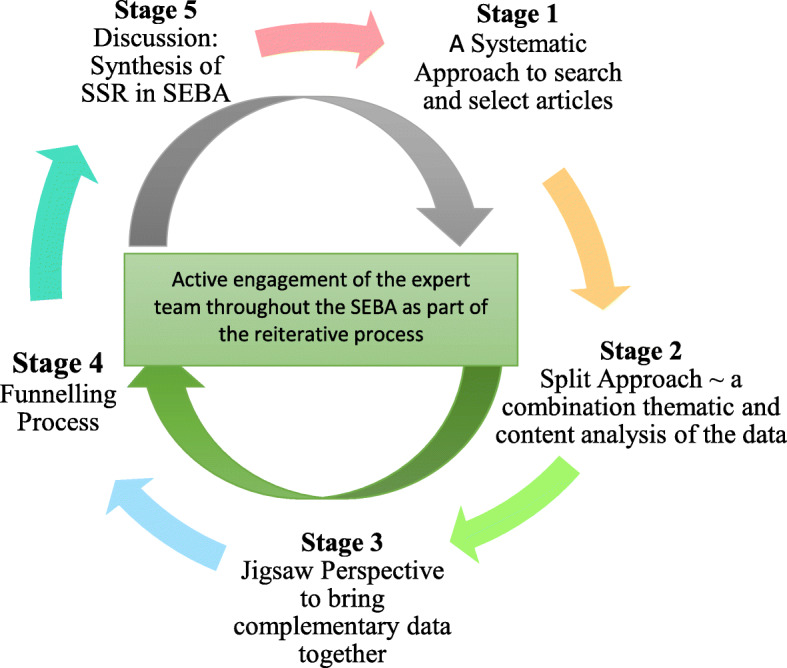
The SEBA Process

Krishna’s SEBA consists of five stages – the Systematic Approach, Split Approach [38, 39], Jigsaw Perspective, Funnelling and SSR in SEBA Synthesis. This process is outlined in Fig. 1.

Diversity of views and the presence of complex individual, academic, research, socio-cultural, professional and personal factors involved in understanding the impact of care of dying patients upon medical students served as the rationale for adopting SEBA as its constructivist and relativist lens allows for the mapping of complex concepts from multiple angles [40–43]. In addition, an interpretivist approach guided the research process.

In keeping with the SEBA methodology, opinions were sought at every stage from an expert team. This team comprised of medical librarians at the National University of Singapore (NUS) Yong Loo Lin School of Medicine (YLLSoM) and National Cancer Centre Singapore (NCCS), as well as local educational experts and clinicians at YLLSoM, NCCS, Palliative Care Institute Liverpool and Duke-NUS Medical School.

### Stage 1 of SEBA: systematic approach

#### Determining review title and background

Together, the research and expert teams identified the overarching goals of the SSR and ascertained the population, context and concept (PCC) to be evaluated [44, 45].

#### Identifying the research question

Designed around the PCC elements of the inclusion criteria, there was consensus amongst the two teams that the primary research question should be *“How does caring for a dying patient affect the medical student – such as in their professional and personal domains and in their perception of self?”* A secondary research question, “*How do medical students react to exposure to dying patients?*”, was also proposed.

#### Inclusion criteria

A PICOS format [44, 45] was adopted to guide the research process as shown in Table 1.

**Table 1:** PICOS

	Inclusion criteria	Exclusion criteria
**Population**	Medical students	• Main focus on other healthcare professionals and other healthcare students ○Doctors ○Nurses, nursing students ○Allied health workers/ healthcare support staff, allied health students ○Other non-medical student populations • Main focus on patients/family/friends ○Patients ○Caregiver, family, relatives, friends
**Intervention/Exposure**	Being involved in care of dying patients	• No involvement in care of dying patients ○No clearly defined patient care experience (e.g. study just explores student attitudes to death/ palliative care) ○Patient population not dying patients (incl. “geriatrics”, patients without specification that they are dying) ○Focus on physician assisted suicide/ medical assistance in death/ suicide ○Focus on organ donation/ transplant ○Personal experience of death of family/ friend • Teaching activities about dying patients without substantial patient care component: ○Simulation/ case-based learning/ hypothetical scenario ○Dissection, prosection, cadaveric studies, autopsy ○Other classroom-based activity ○One-off encounter with dying patient, or non-clinical encounter (e.g. half day experience), as opposed to being part of care team for a substantial duration • Animal studies/ Interaction with animals
**Comparison**		
**Outcome measures**	Impact on medical students’ • Emotions • Attitude • Behavioural changes and adaptations • Personal and professional development • Personal and professional relationships	• Main focus is evaluation and discussion of another outcome: ○Effectiveness of teaching/ assessment methodology ○Student’s performance/ knowledge/ skills ○Patient outcomes ○Others • Evaluation of societal norms, cultural beliefs, acceptability, ethics
**Study design**	• English language • No restriction on design (qualitative, quantitative, mixed) • No restriction on type of publication (includes perspectives, opinion pieces, commentary, case reports, grey literature) No restriction on geographical location of study or publication	• Non-English publications without English translation • Unable to retrieve full article

#### Searching

Three members of the research team carried out independent searches of six bibliographic databases (PubMed, ERIC, Embase, Psycinfo, Cochrane and Web of Science) between 17th November 2019 and 24th April 2020. Only articles published or translated into English between 1st January 2000 and 31st March 2020 were included. These parameters were established in line with Pham et al. [46]‘s recommendations to ensure that the research process would be both viable and sustainable. The full PubMed Search Strategy may be found in Appendix A.

#### Extracting and charting

In order to narrow down the list of full-text articles for review, research team members independently reviewed the titles and abstracts identified from each database. Sambunjak and Straus [47]‘s approach to ‘negotiated consensual validation’ was then employed by the team to collectively arrive at a list for further consideration.

#### Review selection

Research team members then carried out independent reviews of these full-text articles and used ‘negotiated consensual validation’ once again to determine the final list of articles for analysis.

#### Charting the data

Two members then adopted the data charting form designed by Tan et al. [48] to organise all publications by author, year of publication, purpose of review/research question, practice setting, methodology, population characteristics and outcome evaluation.

### Stage 2 of SEBA: Split approach

The research team then split into three sub-teams and simultaneously reviewed the 52 included full-text articles. The first sub-team summarised and tabulated the articles to ensure that all pertinent information was catalogued. Guidelines were drawn from Wong et al. [49]‘s RAMESES publication standards: meta-narrative reviews and Popay et al. [50]‘s “Guidance on the conduct of narrative synthesis in systematic reviews”.

The second sub-team analysed the included articles using Braun and Clarke [51]‘s approach to thematic analysis. The members independently constructed ‘codes’ from the ‘surface’ meaning of the text and located meaningful patterns [52–56] by immersing themselves in the data “without [referencing] any predetermined classification” [55]. A common coding framework was then established and refined at online and face-to-face meetings. Subthemes and themes were then developed upon collapsing the codes into larger concepts. This process yielded a list of carefully delineated themes.

In tandem, the third sub-team analysed the included articles using Hsieh and Shannon [57]‘s approach to directed content analysis. This involved “identifying and operationalising *a priori* coding categories” [58, 59] from Baldwin et al’s [60] paper entitled “Guidelines for evaluating the educational performance of medical school faculty: priming a national conversation”. In keeping with deductive category application, any data not captured by these codes were assigned a new one. The coding categories were also consistently reviewed and revised where necessary. This process yielded a list of carefully delineated categories.

Finally, ‘negotiated consensual validation’ was used as a form of consolidation and peer debrief across all three sub-teams to further enhance the validity of the findings [61].

### SEBA’s reiterative process

As part of the reiterative process, the findings were discussed with members of the expert team. With the prevailing literature suggesting that caring for dying patients affect the very self-concept of the medical student, with ramifications on their personal and professional domains [11–18], significant consistencies were identified with Krishna and Alsuwaigh (2015)‘s [62] concept of the Ring Theory of Personhood (RToP) [63–74]. As such, following discussions between the expert and research teams, the RToP was adopted to guide the research study.

### Theoretical framework

#### Ring theory of personhood

**Fig. 2:**
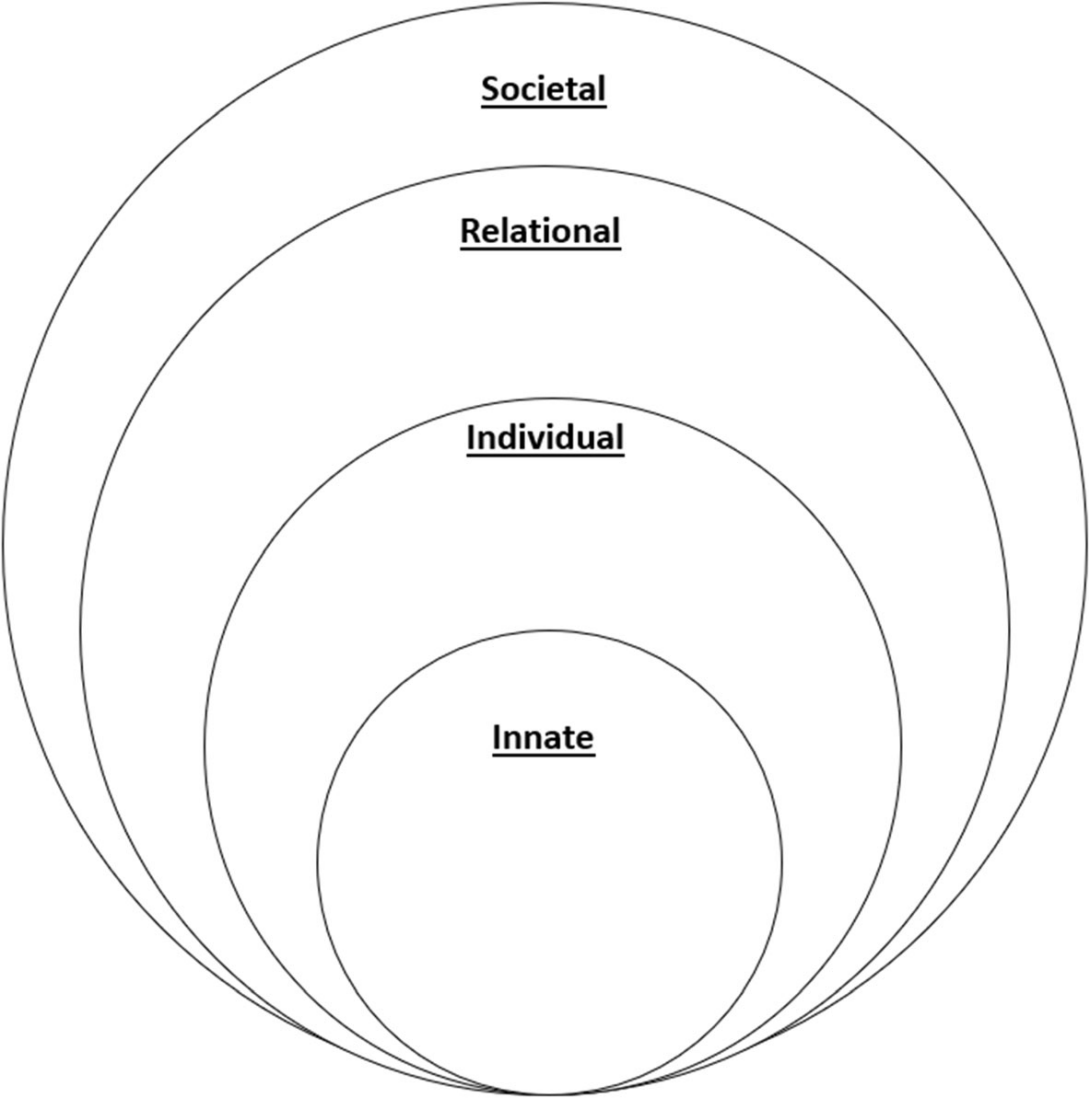
The Ring Theory of Personhood

The concept of personhood or “what makes you, you” put forth by Krishna and Alsuwaigh’s RToP may be described in terms of four domains represented by the Innate, Individual, Relational and Societal Rings (Fig. 2).

The Innate Ring has two components — a Core and the Secondary Elements. The Core of the Innate Ring is anchored on the notion that all humans are deserving of personhood, “irrespective of clinical status, culture, creed, gender, sexual orientation, religion, or appearance”, simply as a result of living and having human physical characteristics [62]. These aspects are unchanging and are retained till death. The Secondary Elements are the elements a child is born into and includes the family and community, their beliefs, values and culture. This component of the Innate Ring, unlike the Core, is alterable.

The Individual Ring is defined as the unique characteristics of a person, such as one’s values, beliefs, goals, personality and character traits, as well as higher order abilities related to consciousness and cognitive function.

The Relational Ring consists of all close, important and reciprocal relationships and may include family and close friends. These ties are determined by the person and may change over time.

The Societal Ring is the outermost ring and encompasses less significant and more impersonal relationships. These include acquaintances and colleagues. Additionally, the ring encompasses societal, religious, professional and legal expectations that guide and police conduct within one’s society.

In adopting the RToP as a theoretical framework amidst suggestions that witnessing death and dying would have significant impact upon the personhood of medical students, the expert team opted to carry out a second analysis of the data using Hsieh and Shannon’s directed content analysis. Codes and categories were drawn from Krishna and Alsuwaigh’s *“Understanding the Fluid Nature of Personhood — The Ring Theory of Personhood”*. In addition, the expert team suggested that this analysis should be carried out by a separate group of researchers to independently verify the idea. As a result, five new researchers were recruited and trained to use this analytical approach.

## Results

**Fig. 3:**
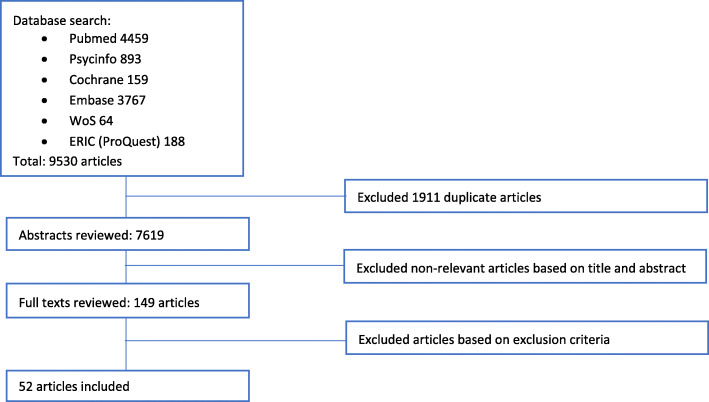
PRISMA Flowchart

Seven thousand six hundred nineteenI abstracts were identified from six databases, 149 articles reviewed, and 52 articles (including 33 peer reviewed articles and 19 grey literature articles) were included as shown in Fig. 3 in the form of a PRISMA Flow Chart [75]. Tabulated summaries of the included articles may be found in Appendix B.

### Stage 3 of SEBA: jigsaw perspective

The jigsaw perspective saw similarities between the themes and categories compared and complementary elements pieced together to form a cohesive picture. It also ensured that critical aspects of the data were not lost when the Split Approach was performed.

### Stage 4 of SEBA: the Funnelling process

**Fig. 4:**
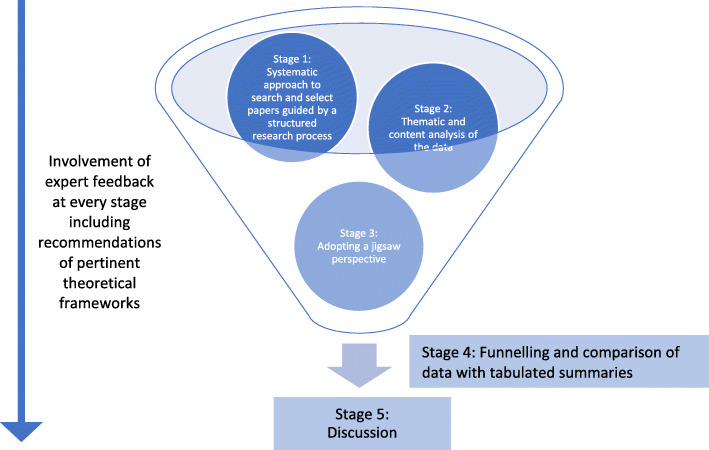
Novel structured approach to SSR

Through ‘funnelling’, themes and categories delineated were compared with key insights from the tabulated summaries to further ensure a holistic picture of the data with minimal overlaps (Fig. 4).

### Themes and categories identified

Scrutiny of the findings by the expert and research teams found that themes and categories from the thematic and content analysis were consistent with one another. To avoid repetition, we discuss the themes identified using both approaches in tandem. The four themes identified were the impact of death and dying on the medical students’ emotional, psychological and behavioral disposition; their attitudes; their interpersonal relationships, and their personal and professional development. These themes are consistent with the RToP framework. As a result, we present our findings through the lens of the RToP, along the four rings.

#### The innate ring

Caring for the dying influences one’s conception of life, death and religion.

#### On life

Many medical students recognised the transitory nature of life [76, 77] and expressed a greater appreciation of its value and the desire to make the most of it [78, 79].

#### On death

There were also personal reflections presented in the included articles on the meaning [80] and concept of death [79, 81–84]. Some reported discomfort and fear when confronted with their own mortality [17, 18, 82, 85] whilst others accepted death as a normal [13, 81, 86], natural part of life [13, 17, 18, 76, 79, 82, 87–89] and appreciated the notion of a “good” death [12, 14, 90, 91]. In addition, most did not see the patient’s death as a failure on the part of the medical team [14, 79]. While some developed a positive outlook [11, 18], some maintained an opposing stance as they viewed the role of medicine as fundamentally life-giving and sustaining [14, 16].

#### On religion

Whilst it would be prudent not to overgeneralise such findings, one study revealed that the experience of caring for dying patients reinforced the students’ religious beliefs [18] and two noted that it enabled them to find meaning in their experiences [18, 92]. However, conflicts may arise when institutions or patients do not share their beliefs [80], as seen in Smith-Han et al. [13]‘s account of medical students realising that bodies were not always treated as sacred in clinical institutions.

#### The individual ring

The impact of death and dying on the Individual Ring may be perceived in how medical students think, feel and act from both a personal and professional standpoint.

#### Personal

Memorable, powerful, inspiring and transformative [16, 78, 81, 85, 86, 93, 94] were some of the descriptors used by medical students when asked to describe their first experience with a patient’s death. For some, caring for the dying was a satisfying experience [16, 17, 88] with some feeling moved [17, 92, 94], humbled and grateful for the opportunity [92, 94–97]. Some students felt more comfortable discussing death and dying after these personal encounters [78, 80, 86, 91]. They also reported being better able to manage their emotions and cope [11–13, 21, 84, 89, 94, 98, 99]. Indeed, two studies reported improvements in the medical students’ management of sadness, hopelessness, and helplessness [18, 100]. Positive coping strategies such as reflective writing [77, 79, 80, 84, 85, 94, 99, 101, 102] were often used to help regulate their emotions [13, 21, 99, 102]. Others sought comfort by partaking in religious rituals or prayer [12, 14, 18, 99], exercise or hobbies [12, 13] or simply by taking time off work [14, 101].

These close interactions taught medical students important lessons on the power of listening [17] and “bearing witness to another’s suffering” [100]. One article found that it instilled humility in the students [103] and encouraged them to reflect on their values [85, 87, 92]. Those who cared for the dying during medical school were also found to have a more positive attitude towards these patients [11, 104], with a greater sense of relief, peace and acceptance of their abilities and limitations [76, 78, 85, 105]. This may in turn minimise rates of compassion fatigue and burnout [94]. Two studies reported that students developed a greater interest in their patients’ holistic medical, psychosocial and spiritual well-being [18, 100].

However, some medical students found themselves breaking into tears [13, 14, 21, 76, 77, 81, 84, 87, 94, 98, 99, 101, 106] and others fighting back their own emotions [81, 84, 102]. Some withdrew by physically stepping away from the situation [87, 94] or isolating themselves [77]. One student defaulted to the reciting of medical protocol while others described “freezing up” [16, 22, 79, 87]. Shortly after their encounter, some medicals students described being in a complete daze [79, 103] or preoccupied with lingering thoughts of the patient [21]. Some expressed their initial denial [18, 77, 99] and envisaged a different outcome [79] while others tried to rationalise their thoughts away [87, 106]. For some, sleep eluded them [21]. For others, vivid imagery and flashbacks [16, 18, 21, 87, 107] incited feelings of distress and persisted for a significant duration after the encounter.

Often, shock [12, 13, 16, 21, 22, 87, 99, 101, 108], confusion and conflict [21, 22, 77, 79, 80, 85, 101–103, 109] were also experienced by the medical students. Being unable to find the “right words” to verbalise their feelings left many “traumatised” [14, 16, 18, 78] and emotionally overwhelmed [18, 21, 81, 84, 85, 87, 92, 98]. In one article, Slim [79] narrated his struggle of reconciling his patient’s “Do-Not-Resuscitate” order with his own desire to “do no harm”. Medical students also reportedly experienced sadness and grief [12, 16, 21, 77, 78, 84, 87, 92, 101, 105, 107, 110], guilt [11–14, 16, 18, 21, 77, 78, 87, 98, 101, 105], anger and frustration [14, 18, 21, 84, 87, 90, 92, 101, 104], a sense of injustice [18, 90, 101] and helplessness at being unable to change their patient’s outcome [18, 21, 22, 78, 81, 92, 97, 99, 104]. A minority described experiencing physical reactions such as throat tightness [76] and paresthesia [21] in the wake of their patient’s death.

#### Professional

When armoured with experience, some medical students described feeling more comfortable [12, 83, 91, 111], confident and prepared for managing their dying patients [12, 16, 17, 80, 85, 86, 89, 92, 97, 110, 112] and better understood the responsibilities involved in processing the formalities of death [13, 87]. Many developed a deeper appreciation of the impact of death and dying on patients and their families [78, 80, 84, 93, 96] and the need for the former’s [17, 85, 88, 93, 95, 96, 98, 99, 104] and latter’s holistic care [21, 92, 103]. Students also began to more consciously view their patients as fellow persons instead of apprehending them by their disease [17, 78, 81, 84, 85, 92, 98–100, 103].

These experiences assisted in their professional identity formation as well [12, 13, 109]. Students were given the opportunity to hone their communication skills [78, 83, 85, 86, 93, 96, 100, 104, 113–115] which led to newfound self-confidence in their clinical role [99]. Witnessing a patient’s death allowed some students to develop greater empathy and sensitivity towards the dying [82, 113]. As opposed to their previous uncertainty and anxiety, some were more self-assured as to what empathetic practice meant [84]. Crawford and Zambrano [89] observed that junior doctors trained earlier in palliative care had enhanced levels of professionalism, communication, teamwork, self-awareness and skills in patient-centered medicine – including attunement to their psychosocial and spiritual needs. Students who cared for dying patients during medical school were also found to have higher knowledge scores on end-of-life care issues [81, 104].

Yet, some medical students also reported suppressing their feelings and detaching themselves emotionally [13, 18, 21, 92, 99, 106] especially in front of their superiors [14]. Over time, they began to “medicalise” their thoughts on death and became increasingly desensitised to the profound humanity of their patients [18, 99].

#### The relational ring

In several medical students, caring for dying patients triggered memories of personal bereavement [16, 21, 84, 87, 106]. Strong emotions were particularly evoked when the patient belonged to a similar age group to their loved ones [12, 16, 21, 82, 84, 87, 116]. As a means of coping with these emotional challenges [12, 16, 21, 86, 89, 98], a number of medical students relied on their own family members for support [12–14, 16, 18, 21, 79, 84, 86, 90, 98, 101].

#### The societal ring

The experience of death and dying had varied effects on the relationship medical students had with members of their Societal Ring – these include their patients and loved ones as well as other healthcare professionals and the profession itself. Broadly, the effect may be classified as either weakening or strengthening.

#### Relationship with patients

- **Weakening:** Some medical students faced difficulty understanding their patients’ perspectives and feelings [84, 95]. Others felt awkward interacting with the dying [22, 80, 85, 117] and were uncertain about their role when doing so [107].
- **Strengthening:** Conversely, some felt that the experience allowed them to better understand the needs of their dying patients [77, 78, 84, 95, 96, 103]. They learnt how to better listen and provide support [12, 14, 17, 84, 97, 99, 100], honed their soft skills in communication [95, 116] and developed virtues such as patience [17] and compassion [82]. Many built rapport, developed attachments [12, 14, 76, 80, 98] and were inspired by their patients’ and their own experiences [86, 94, 108].

#### Relationship with patients’ loved ones

- **Weakening:** Some medical students found it emotionally challenging and stressful to interact with their patients’ loved ones [14, 18, 21, 118], especially if it involved breaking bad news [18] as they dreaded having to deal with the emotional anguish [110]. Pessagno et al. [14] noted that some were also worried about potential litigation issues. At times, the students struggled to reconcile incongruences with their patient’s wishes, that of their loved ones and professional medical opinion their best interests [79, 106]. Some felt pressured to accede to their family’s demands [106].
- **Strengthening:** While caring for their patients, medical students also learnt to communicate sensitively and build rapport with their patients’ loved ones [12, 21, 93, 95, 98]. They learnt the importance of showing empathy and supporting the family through the process [12, 17, 21, 80, 95, 98, 99, 107] and some also journeyed together with them through prayer [98, 99].

#### Relationship with other healthcare professionals and the profession

Individual studies attributed the varied effects on medical student-clinician relationships to different levels of emotional sensitivity and personalities of the clinicians [102], different care settings — emergency department versus inpatient service [21] — and cultural or societal norms in different countries [102].

- **Weakening:** Many medical students struggled with a lack of support and guidance from their seniors and faculty [16, 22, 87, 101, 102, 107]. Some did not feel comfortable approaching their superiors for help. Diverse reasons include the fear of being burdensome [101, 107], their feelings of awkwardness [79], the desire to appear professional [14], the medical team’s insensitivity or lack of emotion [16, 21] and their disagreement with advice proffered by their seniors to simply desensitise themselves to death [77, 102]. Others felt disempowered and discouraged from actively participating in the care of the patient [12, 99, 102]. Such experiences may have contributed to their belief that their educational needs were not adequately met [16].
- **Strengthening:** However, some medical students found comfort in discussing their experiences and emotions with other senior clinicians [12–14, 16, 90, 99, 102]. Some were impressed and regarded their seniors as good role models to emulate their behaviour on [12, 17, 21, 82, 84–96, 99, 102, 113, 118] and were able to built strong positive relationships with them [12–14, 84, 86, 90, 98, 99, 102].
- **On palliative care and the role of doctors:** Medical students became more aware of the value of palliative care and adopted positive attitudes towards it [11, 101]. Baumrucker and Woods [96] reported that medical students felt more comfortable referring their patients with terminal illnesses to hospices. Kearsley and Lobb [84] found that prior negative impressions of palliative care were positively altered. This may be attributed to their broadened understanding of what it means to be a physician — from trying to “cheat death” and prolong life, to preserving their patient’s quality of life and helping them transition towards a more dignified death [13, 17, 92]. Perceptions of the doctor as a life-saving hero was altered to one centred on showing care through the effective management of illness [13] and the provision of bereavement aid [11, 111]. Individual studies reported that through these experiences, medical students acknowledged the limitations of medical intervention [14] and recognised that non-medical acts such as providing a listening ear or a warm embrace may provide much needed healing for the dying patient [76].

### Stage 5 of SEBA: synthesis of SSR in SEBA

The SSR produced was guided by the Best Evidence Medical Education (BEME) Collaboration guide [119] and the STORIES (Structured approach to the Reporting In healthcare education of Evidence Synthesis) statement [120]. In addition, two members of the research team employed the Medical Education Research Study Quality Instrument (MERSQI) [121] and the Consolidated Criteria for Reporting Qualitative Studies (COREQ) [122] to evaluate the quality of quantitative and qualitative studies included in this review respectively (Appendix B).

## Discussion

In mapping how medical students are affected by their exposure to death and dying, this SSR in SEBA highlights the advantage of using RToP as a wider framework to analyse these findings.

### Implications of the entwined rings of personhood

#### The entwined nature of the rings of the RToP

The four rings of personhood do not stand in isolation to one another but are dynamically entwined as originally put forth by Krishna and Alsuwaigh. Whilst the **Societal Ring** is traditionally seen as a means of ensuring that basic standards of practice, etiquette, rights and codes of conduct are adhered to, senior clinicians have an immediate effect on how the medical student thinks, feels and behaves through the provision of timely personalised advice and feedback, role-modelling, support for their professional identity formation, active facilitation of their continuous learning, guidance in the development of their self-efficacy in caring for the dying, and by helping them develop better methods of coping in the face of their patients’ demise [77, 79, 80, 84, 85, 90, 94, 99, 102]. These may be best understood as ‘organisational influences’ which are intrinsic to the medical program’s culture and structure. Well-supported students are inclined to see these trying experiences as transformative [76, 78, 85, 105] and affirmative of their career choices.

#### Building resilience

Such experiences underscore the impact of positive and congruent experiences on building one’s resilience. This highlights a further feature of the RToP, that experiences in one ring may strengthens the others. For example, societal and familial support and religious beliefs that positively impact the **Societal, Relational** and **Innate Rings** also bolster the **Individual Ring.** This helps to build resilience in medical students and boost their self-assurance.

When medical students face challenges in their line of work, significant family and friends from the **Relational Ring** may serve as a prominent source of encouragement, allowing for their reprieve and reinvigoration [12, 21, 84, 87, 116]. Similarly, the reevaluation and reinforcement of their religious and spiritual values within the **Innate Ring** may allow students to derive meaning from and make meaning of their bleak experiences [13, 17, 18, 76, 79, 82, 87–89].

#### ‘Dyssynchrony’

**Fig. 5:**
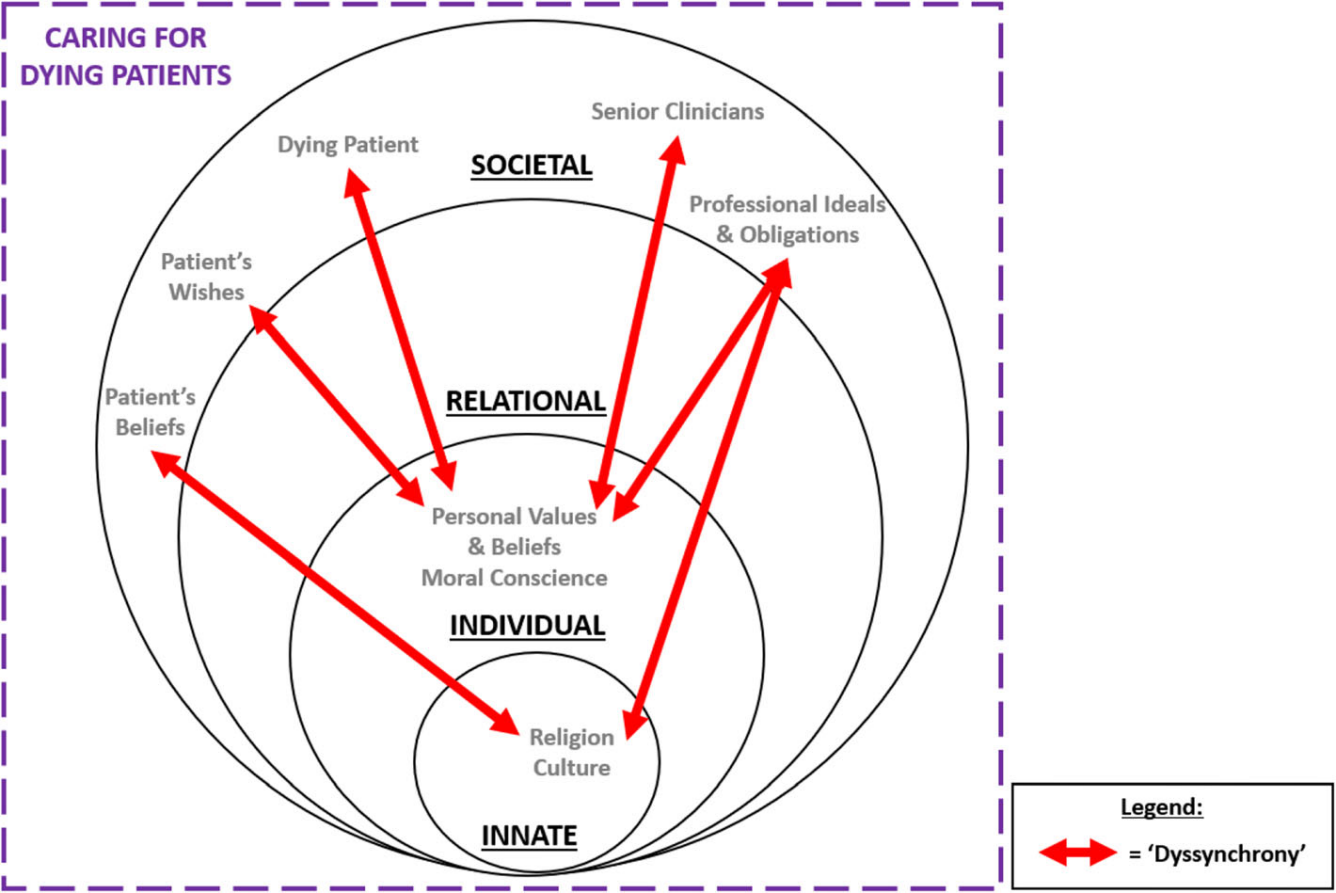
Caring for the dying provokes ‘dyssynchrony’ (red arrows) between the Rings of Personhood

Conversely, evidence of their entwined nature provides a unique opportunity to observe how caring for the dead and dying may result in conflicts or ‘dyssynchrony’ between the rings of personhood. This may arise when changes in one ring run against convictions, values or practices held in another ring (Fig. 5).

Dissonance have been reported between the medical student’s:

1. **Individual** and **Societal Rings**.

- personal beliefs, expression of feelings and expectations that a professional should remain detached and emotionally distant towards patients [13, 16, 84, 101, 102, 107].
- personal values of honesty and the professional ideal of diplomacy and sensitivity [116].
- personal, religious and/or moral duty to save and prolong lives which are “at odds” with the patient’s wishes and professional obligations to respect “Do-Not-Resuscitate” orders [79].

2. **Innate** and **Societal Rings**.

- innate or cultural view that death is a failure and the patient’s subsequent death [16, 79].
- innate view that “young deaths” are unnatural and their occurrence in reality [14, 18, 87].
- religious beliefs about the sanctity of the human body and the attitude adopted towards bodies in hospitals [13].

The ramifications of unresolved ‘dyssynchrony’ between two or more rings may exacerbate and prolong feelings of moral distress and confusion in medical students [18, 89]. Risking potential compromise of their responsibilities as healthcare providers, these feelings may manifest in the form of guilt [18], anger [18, 89], feelings of incompetence [16, 18] and in questioning their “purpose of being a doctor” [18].

In addition, medical students may be conflicted between their obligations to the safety of their families and their duty to augment healthcare workforces in the face of the COVID-19 pandemic [123–128]. The personal and professional desire to always ‘do no harm’ is also compromised amidst reports of feeling overwhelmed and exhausted by the increased workload and shortage of personal protective equipment (PPE) [129, 130].

Higher volumes of dying patients also intensify the dyssynchrony between their aspiration to save lives and their forced reality to let die. Students may find themselves entangled in “emotionally and ethically fraught resource-allocation decisions” [24] due to the utilitarian shift away from individual choice and autonomy, and towards “saving as many lives” [131].

**Fig. 6:**
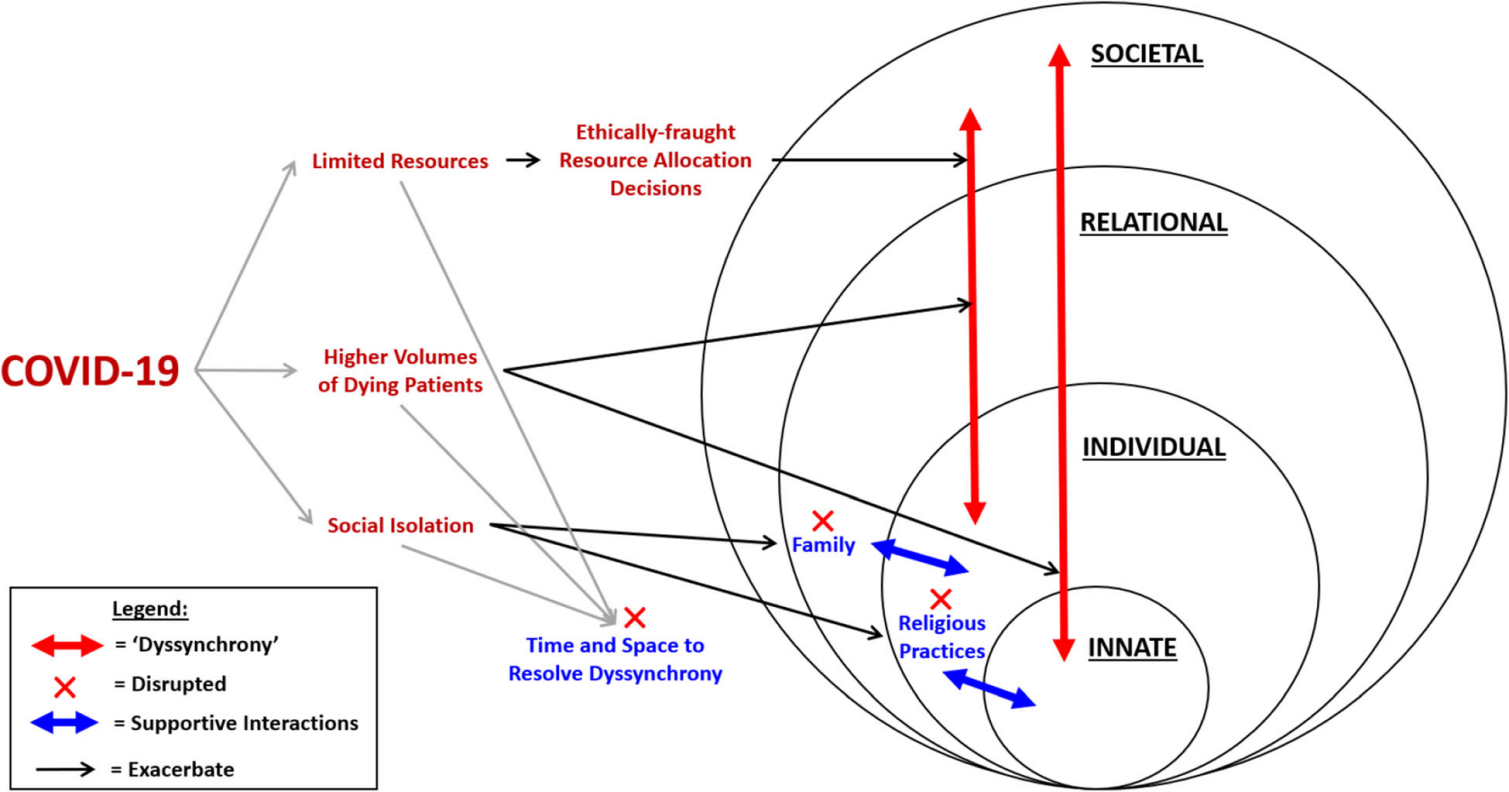
‘Dyssynchrony’ between the rings of personhood exacerbated by the COVID-19 pandemic

These situations are further exacerbated by the act of being thrust into a “completely new context” with the new environment in the isolation wards bringing with them “a sense of oppression” [130]. Restrictions placed on religious congregational services, limited access to usual support systems [132–135], and the discontinuation of death rituals as a result of safe distancing measures may lead to disenfranchised grief, with little time and space to resolve this ‘dyssynchrony’. It could be surmised from the SARS epidemic [136, 137] that such unresolved ‘dyssynchrony’ across the various domains of personhood may result in higher rates of psychiatric morbidity, burnout and post-traumatic stress (PTS) [133–135]. The dyssynchronous effects of COVID-19 across the various rings and the disruptions they bring are presented in Fig. 6.

#### Supportive interventions in medical school curriculum

Evidence of adverse clinical, psycho-emotional, spiritual and personal repercussions underlines the need to consider organizational strategies to manage these risks. While medical students have a potential role in alleviating manpower shortages, this must be weighed against other important considerations such as their physical and emotional well-being, which institutions have a duty to ensure, as well as their potential threat and actual benefit to the system. Their manifold likelihood of carrying and transmitting the virus may “introduce unnecessary risks for patients and other clinicians” and the activation of these students may consume already strained supplies of PPE [104]. Should the organisation be unable to provide adequate support to the medical students and address these concerns, it would not be ethically justifiable to involve them in patient care during this period.

**Fig. 7:**
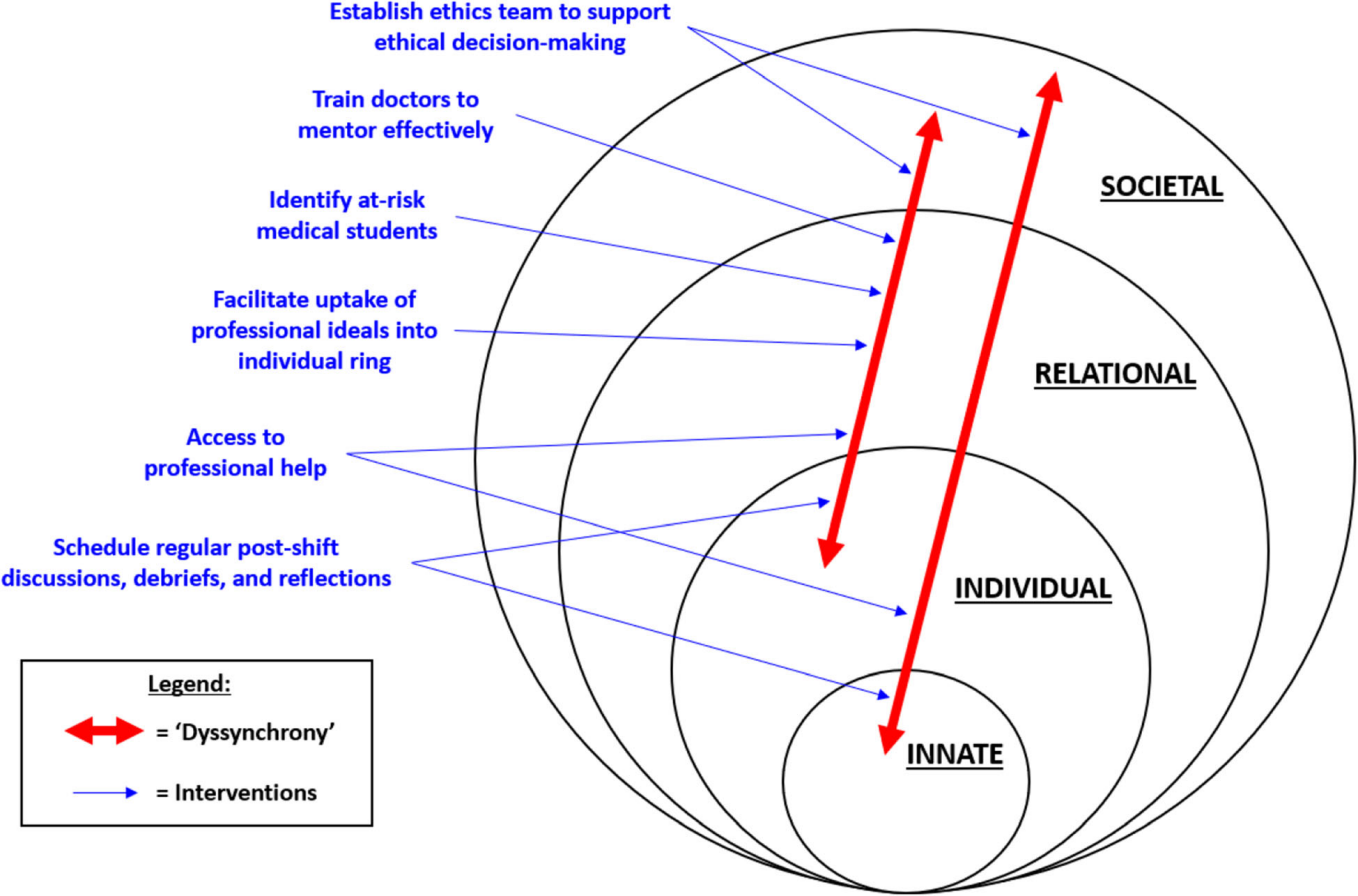
Suggested interventions (in blue) and their target(s)

In light of these and drawing from lessons learnt in ‘peace time’, we proffer suggestions as to how to address the needs of medical students entering and/or returning to clinical care. A phased return to clinical practice is crucial. This will provide educators with the opportunity to establish an ethics team to guide difficult ethical decision-making, train senior clinicians to mentor more effectively, identify at-risk students, facilitate their professional identity formation, offer access to professional help and formally integrate debriefs, discussions and reflections into the curriculum structure (Fig. 7). These interventions are expanded upon in Table 2.

**Table 2:** Suggested Interventions to Improve Medical Students’ Management of Dying Patients

Findings and Problems Faced by Medical Students	Intervention
Clinical attachment with direct interaction with dying patients is an effective way to learn [86].	**Integrate direct clinical care of dying patients into mandatory curriculum** [11]**.**Actively encourage **interdisciplinary and interprofessional collaborations** with nurses, medical social workers, pharmacists and other healthcare professionals who bring with them unique experiences and insights into the care for the dying and their families [137–141]
Lack of debrief, death acknowledgement, and closure. Need for psychological support.• “Often being ‘on their own’” [107].• “Little or no time for discussion or reflection on patient’s death” [16, 101, 118].• “Experiencing ‘a small form of PTSD’ every time he thought of a patient’s death for several weeks after it happened, because no one on his team had acknowledged it.” [107].	• Schedule routine [16] **reflective discussions** (E.g. Focus group discussions) and **debriefs** [16, 18, 21, 22, 78, 89, 101] with clear guidelines [90], as well as after every death including rounding on those who died.• Provide **counselling and access to a psychologist** to medical students who require more support [18, 21].• Incorporating **death rounds** into attachment programs. • Provides an opportunity to explore strong emotions that arise from caring for dying patients with colleagues in a supportive environment.
Medical students found it difficult to address and reconcile conflicts in personhood:• Dilemma of being professionally detached yet still able to display empathy and care towards patients [13, 87].• Conflict between personal values and professional ideals.• Belief that patients under the care of doctors should not die.• Conflict between non-maleficence and having to triage decisions [24, 134, 142].	• **Facilitate uptake of professional ideals** into individual ring of personhood. • Minimizes ‘dyssynchrony’ between the rings of personhood leading to newfound self-confidence and empowerment [84].• Incorporate **discussions** of professionalism in palliative care.• Provide advice and standards on how best to calibrate emotional attachment in the care of dying patients, and also to balance seemingly conflicting ideals.• Encourage medical students to express any internal conflicts they have during **debriefs**, **death rounds**, or privately with a trained **mentor**.• Establish **ethics teams** to support and be consulted on ethical decision making.
Inconsistent or weak medical student-doctor relationship with lack of support and guidance [16, 22, 87, 101, 102, 107].• Felt seniors were not ideal role models [102].• Gave conflicting accounts of professionalism [102].• Felt disempowered and discouraged from actively participating in the care of the patient [12, 99, 102].• Some medical students feel uncomfortable approaching superiors for help [14, 16, 21, 79, 101, 107].	**Train doctors to mentor effectively:**• Role model skills, such as communication with the dying [78], through explicit demonstrations [12].• Routinely inquire of trainees about their and acknowledge their feelings [16].• Discuss and attend to emotional aspects of death with team [16].• Observe medical students and provide feedback [114].• Provide a standardized guide of professionalism milestones.• Provide a safe learning environment.• Identify medical students who need support.• Train healthcare workers to spot signs of psychological distress in their colleagues.
The experiences, reactions, and preferred support systems of medical students to be varied.• While some preferred to seek support from within their relational ring [12, 100], others preferred to turn to peers and clinicians from their societal ring [12, 14, 86, 90] possibly because of the shared experience amongst members of the medical community [12, 14] that made them feel better understood [14].	**Adopt a tailored approach to intervention to individualize support for medical students**.• Ensure different options are available to medical students to help cope.

### Limitations

This review is not without its limitations. This SSR is limited by articles published in English or with English translations. Hence, much of the data comes from North American and European Western countries or in the English language, skewing perspectives and raising questions as to the applicability of these findings in the setting of other cultures. Whilst databases used were selected by the expert team and the team utilized independent selection processes, some critical papers may still have been omitted. Despite the use of the Split Approach and tabulated summaries which allowed for triangulation and transparency in the direction of the SSR, reviewers’ inherent biases could still have an impact on the data analysis. Furthermore, while quality assessment of included articles was conducted using MERSQI and COREQ, we were unable to quality assess all the articles due to the heterogeneity in the methodologies used. While many supportive interventions were identified in this review, this SSR was not designed to assess them. More evidence-based literature reviews are required to examine the effectiveness and extensiveness of supportive interventions. As we used a single model (RToP) to review the impact of death and dying on medical students, imperfections and presumptions from the models are transferred to this review. As such, studies employing other models of personhood can be integrated to support our findings.

## Conclusion

The findings of this SSR in SEBA should be a rallying cry to ensure that medical students are effectively supported. It is clear that support of trained senior clinicians who are sensitive to the dilemma and conflicts that students working in a structured and nurturing environment is key in the era of the COVID-19 pandemic and beyond. The silver lining in these unprecedented times may be a chance to correct years of poor preparation. We have much to learn but the adversity posed now may be just the impetus to make the change.

## Supplementary Information

**Additional file 1: Appendix A**. PubMed Search Strategy

**Additional file 2: Appendix B**. Summary of Included Articles

## Data Availability

All data generated or analysed during this review are included in this published article [and its supplementary information files].

## Abbreviations

COVID-19: Coronavirus Disease 2019
SSR: Systematic Scoping Review
NUS: National University of Singapore
YLLSoM: Yong Loo Lin School of Medicine
NCCS: National Cancer Centre Singapore
PCC: Population, Concept and Context
PICOS: Population, Intervention, Comparison, Outcomes, Study Design
RtoP: Ring Theory of Personhood
MERSQI: Medical Education Research Study Quality Instrument
COREQ: Consolidated Criteria for Reporting Qualitative Studies
BEME: Best Evidence Medical Education
STORIES: Structured Approach to the Reporting in Healthcare Education of Evidence Synthesis
PPE: Personal Protective Equipment
SARS: Severe Acute Respiratory Syndrome
PTS: Post Traumatic Stress
