# Supplementary material for: The impact of death and dying on the personhood of medical students: a systematic scoping review
Source: BMC Med Educ. 2020 Dec 28;20:516. doi: 10.1186/s12909-020-02411-y (PMC7768997; doi:10.1186/s12909-020-02411-y)
Supplement: Supplementary file 1 — Additional file 1: Appendix A. PubMed Search Strategy [file 12909_2020_2411_MOESM1_ESM.docx]

**Appendix A – PubMed Search Strategy**

OR

AND

AND

|  |  | MeSH | Keyword |
| --- | --- | --- | --- |
| Population | Medical stude  nts | "Students, Medical"[Mesh]  "Education, Medical, Undergraduate"[Mesh] | “Medical student”[tiab] OR “Medical students”[tiab]  (medical[tiab] OR medicine[tiab] OR clinical[tiab]) AND (student[tiab] OR students[tiab]) |
| Intervention / Exposure | Being involved in care of dying patients | "Terminally Ill"[Mesh] OR "Critical Illness"[Mesh] OR "Death"[Mesh] OR “Catastrophic Illness” [Mesh]OR "Life Support Care"[Mesh] OR "Critical Care"[Mesh] OR "Palliative Care"[Mesh] OR “Terminal care” [Mesh] | dying[tiab] OR terminal*[tiab] OR critical*[tiab] OR “life threatening”[tiab] OR palliative[tiab] OR “end of life”[tiab] OR “end-of-life”[tiab] |
| Comparison / control |  |  |  |
| Example outcome measures | 1. Emotions | Emotions[MeSH] OR "Stress, Psychological"[Mesh] | grie*[tiab] OR sorrow*[tiab] OR bereave*[tiab] OR  sad* [tiab] OR distress[tiab] OR stress*[tiab] OR burnout[tiab] OR emotion*[tiab] OR fulfil*[tiab] OR inspir*[tiab] OR satisf*[tiab] |
|  | 2. Attitude | Attitude[MeSH] OR Motivations [MeSH] | attitude*[tiab] OR motiva*[tiab] OR incentiv*[tiab] OR perspective* [tiab] |
|  | 3. Behavioural changes and adaptations | “Defense mechanisms” [MeSH] OR “Adaptation, Psychological” [MeSH] OR Behavior [MeSH] | “defensemechanism” [tiab] OR adaptation [tiab]OR behaviour* [tiab] OR performance [tiab] OR conduct [tiab] |
|  | 4. Personal/ professional  development | "Clinical Competence"[Mesh] | “personal development” [tiab] OR “personal growth” [tiab] OR “professional development” [tiab] OR “professional growth” [tiab] |
