# Supplementary material for: The impact of death and dying on the personhood of medical students: a systematic scoping review
Source: BMC Med Educ. 2020 Dec 28;20:516. doi: 10.1186/s12909-020-02411-y (PMC7768997; doi:10.1186/s12909-020-02411-y)
Supplement: Supplementary file 2 — Additional file 2: Appendix B. Summary of Included Articles [file 12909_2020_2411_MOESM2_ESM.docx]

**Appendix B – Summary of Included Articles**

| **Author, Year** | **Title** | **Background** | **Theoretical Approach and Methods** | **Main Empirical Findings** | **Insights drawn** | **MERSQI (Max 18)** | **COREQ (Max 32)** |
| --- | --- | --- | --- | --- | --- | --- | --- |
| S. S. Carmody, R. M. Arnold, J. Wohnsiedler, R. Schuh and D. Barnard 2002 | Attitudes of medical students toward working with dying patients | Society is now more interested in end of life care and there is a growing need to address that by training physicians to provide excellent palliative care | 1^st^ year and 2^nd^ year graduating students at University of Pittsburgh were surveyed regarding their knowledge, attitudes and training in end-of life care  7 4-point Likert scale questions selected from the Attitudes about Care at the End of Life Scale to Elicit student’s attitudes working with dying patients.  A conference abstract | Of the overall response rate 382/443 (86%), respondents were 31% 4^th^ year, 35% 2^nd^ year and 34% entering students.   1. There was no difference in attitudes based on race 2. More women than men felt psychological suffering can be as great as physical suffering 3. 4^th^ year students dreaded dealing with dying patients family member’s emotional distress less and felt less guilty about patient deaths | Students who viewed themselves as more inclined to the social and emotional aspects of medicine were more likely to have attitudes that favour working with patients at the end of life.  Personality characteristics may be useful in identifying students suited for palliative care careers in their early training | NA | NA |
| R. MacLeod, C. Parkin, S. Pullon and G. Robertson 2003 | Early clinical exposure to people who are dying: Learning to care at the end of life | R. Macleod seeks to discover how to create opportunities for medical students to learn how to care for palliative patients and to understand how medical students learn from dying patients | The approach was not explicitly stated but it seems to imply a thematic analysis with Heideggerian phenomenological perspective (the notions of shared background practices and familiarity with social, cultural and professional norms [such as a group of medical students may have]) was chosen for the analysis. They analysed student’s oral and written reflections about their experience in the hospice  Employed Braun and Clarke’s approach to thematic analysis. | 58 portfolios were available for use but only 51 portfolios were used as the source of data (as the rest were incomplete). There were 120 significant statements abstracted and 15 themes were identified from there but only 5 themes were noted by more than 20% of the students:   1. The experience was different from what the student had anticipated – due to prior knowledge of death as a lay person. 2. Emotional element of the experience – although it was uncomfortable at first, students got intimate with the patient and their families and expressed their views 3. Spiritual and religious elements: students reflected on the reason for “being” and the meaning of life 4. Personal reflections: - help them create new ways on dealing with the situation and synthesise prior medical knowledge in the context of the case 5. Future caring approaches: students identified this by reflection of their values and incorporating them into their actions | Growth of medical students:   - Increased self-awareness by understanding their emotions - Reflection on the meaning of life - Discovering new approaches to caring - increased ability to use medical knowledge when speaking to patients   Dealing with palliative patients at an early stage of their career can influence the care of doctors in the future  There is further need to expand and continue this model | NA | 12 |
| M. E. Billings, R. Engelberg, J. Curtis, S. Block and A. M. Sullivan 2010 | Determinants of medical students' perceived preparation to perform end-of-life care, quality of end-of-life care education, and attitudes toward end-of-life care | M. E Billings seek to discover how conceptual models of learning contributed to medical student’s perceived preparation, competency and attitudes to perform end-of-life care. | Not explicitly stated. A quantitative approach was adopted. Scales for predictors and outcomes were created. A mix of “yes”/ “no”, 4 -point Likert-like scale and 5-point Likert scale was used for different factors which included predictors for formal, informal and hidden curriculum, preparedness, attitudes toward end of life and quality of end of life education.  Scores were summed up and a multivariate regression analysis to investigate the association between each of the outcome variables. | 1455 4^th^ year medical students participated with a response rate of 62%, the refuse rate was 8% and the rest could not be reached. Overall findings: medical students across USA lack comprehensive education about end-of life care.  Curricular scales:   - Formal: Majority said that they have been taught end-of life care - Informal: students report having observed EOL care but not providing it - Hidden: only 20% reported faculty conveying negative messages “a lot”, only 60% reported that faculty felt “rewarded” caring for EOL patients   Outcomes:   - Perceived preparedness: “moderately well prepared” for EOL tasks, least prepared to manage their feeling - Quality of education: majority of students felt their education was “good to very good” - Attitudes: 25% dreaded caring for emotional distress of families and 45% thought that EOL care was depressing   Predictors:   - Formal and informal curriculum were positively associated with preparedness and quality of EOL education - Hidden curriculum was negatively associated with preparedness and quality of EOL - Attitudes were positively associated with informal curriculum, not associated with formal and negatively associated with hidden curriculum | EOL curriculum takes 3 forms: formal, informal and hidden. All are essential and form part of the student’s learning in EOL care. They have different associations with outcomes in students. With role models being the most important aspect of learning EOL care  Holes in EOL care curriculum:   - Less opportunity for practice - Less students received feedback - Negative role models - Negative messages from the hidden curriculum impairs students compassion and confidence | 6.5 | NA |
| E. Borgstrom, S. Cohn and S. Barclay 2010 | Medical professionalism: Conflicting values for tomorrow's doctors | E. Borgstrom recognises that the hidden curriculum needs to change however it is difficult to do so, hence the author looks at the ways in which the hidden curriculum lags behind and how it conflicts with new values underpinning education reform | Not explicitly stated but the paper seems to employ a thematic approach that analyses student portfolios of those who have met EOL care patients, viewing the content of the portfolios as a representative of general values imparted to students throughout their education. Items were analysed during an interpretative approach, striving to find meaning beyond straightforward discrete references.  Employed Braun and Clarke’s approach to thematic analysis. | 234 portfolios of final year students from Cambridge were available for analysis: 116 from the GP and 118 from hospital settings. 3 main themes of conflict emerged:   1. Ethics as a source of tension: pain relief at EOL care, palce of death/care, withholding and withdrawing treatment, patient autonomy 2. Doctor patient interaction: fear of causing patient upset, patient refuses information, confidentiality 3. Managing subjective boundaries: detachment, expressing emotions and situations that bring up one’s personal experiences | Tensions arise due to different values that underlie the concept of professionalism throughout a student’s education (formal and hidden) and not anything specific to EOL care.  Values are embedded in the routines and practices of the organisation, and cannot therefore be simply addressed through redesigning curricula or individuals championing change  However, this tension may be the key to lasting features of medical professionalism. | NA | 16 |
| R. T. Masia, W. J. Basson and G. A. Ogunbanjo 2010 | Emotional reactions of medical doctors and students following the loss of their patients at the Dr George Mukhari Hospital emergency unit, South Africa | R. T. Masia wanted to explore the thoughts and feelings of doctors and medical students who have lost patients while under their care and explore the links between the existing literature, which was deficient, and to add to empirical literature on patient-related bereavement studies. | A qualitative research design following a phenomenological approach was used. There was purposeful sampling – 10 participants (doctors and medical students) who had experience the loss of a patient in the unit preceding 48 hours before the interview was conducted. Individual free attitude interviews were conducted.  Collaizi’s6 method of descriptive analysis was used and the data was then grouped into themes. | 10 participants at the Dr George Mukhari Hospital emergency unit in Ga-Rankuwa, South Africa’s free interviews were used for analysis. Emerging 11 themes:   1. Emotional reactions: hard for doctors, grief, pain, helplessness and mourner’s inability to derive a sense of meaning from the experience of the death 2. Recurrent thoughts about the incidence: painful loss has not yet been resolved, the inability to tolerate emotional distress is exhibited by avoidance 3. Blame: feel responsible 4. Perceived incompetence: feelings of failure, overcome by working harder and longer hours, excessive need for approval and compulsive approach to work 5. Detachment from emotions: put a barricade between themselves and their patients to avoid any feelings that could result from the death to avoid compromising medical judgement, some desensitisation 6. Religion: provide shelter from chaos and the need to make sense of an essentially senseless event 7. Death of a paediatric patient: more tragic than any other death, unprepared. 8. Medical training: training received is not adequate to deal with loss of patients 9. Psychological service: counselling is required to help cope with loss but no doctor has seen a psychologist 10. Work environment: role stress due to role ambiguity, role conflict and role overload 🡪 burnout. Lack of staff and equipment 11. Coping with the family of the deceased: difficult to communicate, families should not be placed in the position of the consoler | A doctor susceptible to a traumatic-grief syndrome.  Increased deaths and recency of death results in increased distress.  Coping strategies: avoidance, denial, detachment, desensitisation and religion  Biggest problem faced: difficult to cope with the families of their patients  Therefore, doctors need enhanced training in communication skills and communicating death to the patients’ families  Bereavement counselling and debriefing ought to be available to provide them with an opportunity to share emotional responses and reflect on the patient’s fatality | NA | 11 |
| R. Jones and F. Finlay 2010 | Medical students experience of paediatric deaths | R. Jones wanted to investigate medical students experience of paediatric deaths. | Not explicitly stated.  Final year medical students at University of Bristol did an anonymous online questionnaire.  A conference abstract | 220 students were contacted with 60% responding. 27% had witnessed a paediatric death.  Students had mixed feelings after death: anger at self and guilt.  More ‘expected deaths’ was experienced in the UK  Formal support was a good coping strategy. But only 13% felt that medical education had prepared them adequately. | Students feel that although they may have “medical knowledge” they are still lacking in emotional support and are often inadequately supported around the time of a patient’s death, especially a child.  Medical schools need to address these deﬁciencies | NA | NA |
| M. Pestinger, M. Bouteleux, L. Radbruch and F. Elsner 2010 | The patient as a teacher-the meaning of death and dying for medical students | M. Pestinger felt that important concerns of patients are not adequately discussed, others found a significant mismatching in assessing patients preferences in end-of-life care by medical interns, as well as attending physicians. Also a majority of medical students feel uncomfortable with dying patients impeding learning about end-of-life care. | Not explicitly stated. A pilot education program was designed for medical students to accompany inpatients at a Palliative care unit for a minimum of 40 hours. Then a written report was made and analysed qualitatively.  A conference abstract | 19 student’s reports were available for analysis.   - There was uncertainty during first contact - Experienced a deep appreciation of patient’s will and perception - Problems: saying good bye to unexpected deaths, how to handle conflicting emotions | It is an important experience to capture the meaning of death and dying within the physician’s role.  Hence, the reflection on the physician’s role are valued as essential together with the process of gaining resources in coping with the strains. | NA | NA |
| A. P. Nguyen 2011 | How palliative care transformed my perspective of medicine | A. P. Nguyen reflects on how following a palliative care doctor has changed the her perspective on medicine. | A personal reflection of a medical student.  The author recounts her personal experience and draws parallels to what was experienced in the wards during her medical education at a palliative care unit. | Her perception of a doctor was challenged.  New insights:   - A doctor must weigh the beneﬁt that a treatment may provide versus the potential burden it may inﬂict on the patient - Perhaps palliative care can help families better prepare for death - A doctor must extend his concern to the family and friends of his patients as well. - doctors do indeed heal and save people but it does not always result in prevention of death - resolve of become a doctor is strengthened when she accepts the inevitability of death | A doctor’s role may not always be one of “healing” and this may be difficult for medical students to accept.  Palliative care aids this process of acceptance and allows students to gain new insights that a doctor heals not only psychical pain but emotional ones as well. A doctor’s care must be extended to the family as well. | NA | NA |
| J. Liao 2011 | A matter of the heart | J. Liao writes a reflection on his experience dealing with a patient’s brother after the patient’s death. | A personal reflection.  The author recounts his experiences with the patient’s elder brother prior to the patient’s death and gains new insights as to the role of patients. This also changed his approach to future patients. | Patient’s roles are not identified by their disease but rather relationships with others. These roles would last long after the patient dies.  Medical routines are more than routines for patients and their families because there is anxiety and hope in each encounter.  In order to help people through death, his heart needs to be exercised, he also has to recognise his own limitations and affirm the highest values in patients lives. | Dealing with the patient’s family after death is not easy. As their relational roles to the patient require attention that the medical student finds it hard to give.  Medical students need to have empathy and humility to recognise limitations so to better cope and manage the patient’s family, | NA | NA |
| E. A. Morell 2012 | Learning that a death can be a good death | E. A. Morell narrates his experience with a paediatric death and reflects on the new insights gained | A personal narrative reflection.  The author recounts his experience with his first paediatric death in the wards as a first year medical student and describes the new insights received. | Prior to the encounter:   - know very little about EOL care - never had exposure to death   During the encounter:   - felt overwhelmed by helplessness - felt inadequate as he did not deserve to be there   After the encounter:   - wondered if the patient had the best possible death - determined to learn as much as he can to alleviate suffering | Doctors must know how to help people through their illnesses not just to cure them.  Compassionate response is caring for patients when medical intervention is futile.  Alleviating suffering is may mean helping the patient to cope with death but this is not something to fear. | NA | NA |
| R. Pessagno, C. Foote and R. Aponte 2013 | Dealing with death: Medical students' experiences with patient loss | The ultimate goal of this study is to better understand how medical  students experience death as they complete their rotations in which they interact with  patients under the supervision of attending physicians. | A qualitative study of semi structured interviews. New questions and revision of old ones was drafted during the data collection. Grounded theory analysis was used. Themes were created. | 20 3^rd^ and 4^th^ year students were recruited for the study – mix of students who had and have not had experience with dying patients in the palliative care setting.  Gross anatomy lab experiences: 3 themes   1. experiences with anatomy lab as an undergraduate: discomfort, emotional detachment 2. experiences with anatomy lab as a medical student: felt discomfort and anxiety when actively dissecting the cadaver, but not expressing discomfort is the norm 3. coping strategies used to manage discomfort in anatomy lab: dehumanising the cadaver (helps to cope), humanising the cadaver (to maintain respect), simultaneously doing both (mix of both at different timings)   Patient death experiences: 2 themes   1. Descriptions of patient deaths    1. Adult deaths: illustrate the human side of medicine and the role that human judgement played on patient care outcomes, having a patient that is unknown to the student does not trigger an emotional response    2. Paediatric deaths: increased distress compared to adults 2. Coping with deaths as a medical student    1. By talking to people they can turn to for support: family, friends and doctors    2. Carrying on with physician care is the norm in the profession – even if a patient just died    3. Participating in Rituals after infant deaths: dressing the baby    4. Crying    5. Turning to religion   Attitudes toward patient death: 3 themes   1. General view of patient death: most students do not think of death as a failure of the medical system, only if death was due to a procedural error will it be considered a failure of medicine. 2. Meaning of a good death: quality of life is considered, free of pain, patient is comfortable, some level of patient autonomy – choose when, where and how the patient wants to die, good familial relationships 3. Attitudes towards physician assisted suicide:    1. Unsupportive: not doctor’s role according to the Hippocratic oath, there should be a speciality for PAS, many statements tried to reconcile the desire to relieve pain and suffering towards the end of one’s life without directly and intentionally ending one’s life    2. Uncertainty    3. Supportive: but only when the patient has a terminal disease | Dissecting a cadaver causes a medical student to face death and they ultimately learn to cope with death. But death is still viewed negatively.  However, after experiencing patient death, their ideas of death have changed from something negative to something positive instead.  Students do have coping mechanism for death that they come up with on their own.  Most students view the role of the doctor a healer and hence doctors should not have the active intent to kill patient. | NA | 15 |
| A. Gadoud, Y. Adcock, L. Jones, S. Koon and M. Johnson 2013 | It's not all doom and gloom: Perceptions of medical students talking to hospice patients | The authors  aimed to explore in detail the views and experience of medical students who had spoken with patients during their hospice placement | Not explicitly stated. A thematic analysis on semi structured interviews.  Employed Braun and Clarke’s approach to thematic analysis. | 26/30 (87%) 3^rd^ and 4^th^ year students indicated that they have seen a patient at a hospice. 20 of these students decided to take part in the study. Data saturation was reached after 14 interviews. There were 4 major themes and some subthemes:   1. Preconceived idea and fears about how they would cope: with the environment, fear of death and worrying about communication skills with patients 2. Hospice placement: memorable patients – taught and inspired students, environment – was lovely despite their expectations of a sad morbid place, staff- welcoming, organization of placement – should have been longer 3. Impact on students: learning about palliative care – understanding core principles, improved skills – communication with patients, how to cope with emotions when seeing patients 4. Advice to future students: make the most of the opportunities | Despite the emotional burden students will face, visiting the hospice was found to be enjoyable and a valuable learning opportunity – patient encounters were inspiring and impactful.  Hospice staff should not be overprotective by decreasing the opportunities for students to meet with patients because students do cope and they need this opportunity to learn.  It is recommended that hospice staff be supportive instead. | NA | 18 |
| B. Kimball 2017 | On healing: A medical student's lessons from hospice | B. Kimball narrates her experience at the hospice and describes the new insights gained through reflection. | A personal narrative reflection.  The author reflects on her hospice experiences contrasting two deaths – one that the family was prepared for and one that was sudden. She then reflects on medical education and comes up with a new resolve moving forward when dealing with EOL patients | First time in the hospice:   - Struck her that it did not feel like a hospital or clinic - It was apparent that this place was for people to spend their last days   First encounter with death: the expected death with a prepared family   - Felt peaceful - Knew it was not her last encounter   Second encounter with death: the unexpected death with a unprepared family   - Felt emotional, almost cried - Learnt that even at EOL, healing is possible - Realised that medical school only taught her how to extend life and not to confront death - Hospice given her a chance to reconnect with the human experience - Acknowledge that life is finite - Hopes to remember these experiences | Experiencing death was necessary for the author to realise that medical school did not teach her how to deal with it.  Hospice provides new insights for students – healing is possible at EOL, unexpected deaths are the toughest to deal with, realising that death has to accepted by the medical profession and understanding that medicine has it’s limits. | NA | NA |
| S. Udeshi and D. Mohess 2018 | Palliative Medicine: A Medical Student's Perspective | S. Udeshi reflects on her experience in palliative care and how it has transformed her understanding of medicine and patients. | A student’s personal reflection.  An incident of a death was recounted and the student shares her insights on the matter. | Insights:   - Important to understand how patients and their families define quality of life - Understand that families can change their mind after a decision is made - Empower patients and their families to make decisions by providing them adequate information - Understand the patient holistically – physical, psychosocial and spiritual | Benefits of palliative care for students:   - Palliative care addresses the questions that students have - Allows patients to become the centre of care - See how palliative care also cares for the family members - Teaches students how to empower patients and their families to make informed choices | NA | NA |
| W. Stecho, R. Khalaf, P. Prendergast, A. Geerlinks, L. Lingard and V. Schulz 2012 | Being a hospice volunteer influenced medical students' comfort with dying and death: a pilot study | The authors aim to measure the effect of an elective pilot hospice volunteer program for first year medical students on participant’s comfort levels with a dying patient in comparison to a control group and to explore participants’ experiences as their relationships with both patients and hospice volunteers developed. | A prospective study using both a quantitative approach and qualitative approach was used. Participants were surveyed quantitatively at 3 points – pre-programme, post hospice volunteer training and post program (after visiting the hospice proper)  Quantitative approach:   1. Collet-Lester Fear of Death Scale (CLFoD), which uses a 5-point Likert scale - Low CLFoD were correlated with increased comfort when speaking with terminally ill people. 2. Hayslip Communication Apprehension Regarding the Dying Scale (HCARD)- Low levels of apprehension, as measured by the HCARD, were associated with positive attitudes toward aging and death and a greater ability to relate to dying persons in an open and honest manner 3. Descriptive statistics and independent t test was calculated 4. Wilcoxon Signed Rank Test was used   Qualitative approach:   1. Focus groups – members of the research team led the focus groups, after one client visit, mid-program and upon completion of the programme 2. Journals – two entries 3. Thematic analysis was performed based on grounded theory methodology | 12 participants randomly selected from a group of applicants. 114 unselected applicants and non-applicants were combined into the control group. Students with previous hospice experience were excluded. (n=3)  Quantitative results:  Preprogramme:   - No significant statistical difference between participants and non- participants   Post training:   - Statistically significant decrease in participants’ CLFoD scores happen between pre and post training - participants’ HCARD scores during the training period were not affected   Post programme:   - There was no significant statistical difference in non-participants pre and post programme score - There was a statistically significant decrease in participants’ average CLFoD and HCARD scores by 18% for both   Qualitative results: 3 main themes   1. Challenges: preconception that dying was a negative experience, formed intimate relationships with patients which resulted in negative emotions but positive emotions were prominent in discussions 2. Learning: from patients, volunteers and standard medical school curriculum, participants appreciated patient’s perspective on treatments 3. Growth: increased comfort with death and dying, patient centred view on dying, awareness of providing EOL care | Hospice volunteer programme led to statistically signiﬁcant decreases in participants’ anxiety regarding death and communicating with dying patients, as measured by two independently developed and validated scales.  Hospice training alone may be effective in reducing death anxiety.  Experiential learning contributes to a sustained decrease in death anxiety and contributes exclusively to reduction in communication apprehension.  Focus group discussions played a role in the student’s learning during the programme as they could freely talk without the influence of medical professionals. | 7 | 14 |
| S. J. Baumrucker and S. Woods 2000 | The personal side of hospice and palliative care education | S. J. Baumrucker writes a commentary of S. Woods’ reflection during her year 4 palliative care posting. | A commentary on a personal narrative reflection of a year 4 student during her palliative care posting. | S. J. Baumrucker’s opinion on medical student reflections:   - Interesting to read a first hand account - Wanted to see if her internal experienced matched his in anyway   S. Woods’ reflection:   - Saw humanity in its steady state – have to consider patient as a whole - Better understanding of pain management - Understood that causes of physical pain and belief of pain treatment could be different for each patient - Caring for physical symptoms was only the tip of the ice berg of hospice - Increased trust in the hospice care system - new positive light in pain and symptom management and end-of-life care | Hospice care can be a gateway for medical students to understand: death in a positive light, how to view the patient holistically, different beliefs and values of patients, role of family and how to change treatment goals to comfort and support rather than intensive life support | NA | NA |
| D. Wear 2002 | Face-to-face with It: medical students' narratives about their end-of-life education | This essay is an exploration of end-of-life issues relevant to medical training and a call for thoughtful curriculum change.  The author has an interest in medical students’ experiences in end-of-life care, particularly what they have learned from such experiences and what they wish their education had provided to them before they cared for dying patients. | A qualitative inquiry, using thematic analysis of student’s personal accounts of their clinical experiences surrounding end-of-life care and the data generated was explored by the author. Excerpts were combined with medical education literature and the literature was used to amplify or interrogate the excerpts. The author also proposes possible reasons to further explain the student’s experiences. | 98 4^th^ year medical students were asked to volunteer in the study via email. 10 volunteered their written accounts.  4 Themes were generated  Student’s worry and uncertainty about end-of-life care:   - From students: lack of experience in dealing with EOL left students shocked/uncertain when they had to deliver bad news to the family on their own, the formal curriculum did not prepare them well for this - From literature: many clinical students have not witnessed a physician talking to a dying patient or discussed EOL care with a physician, physicians themselves find it hard to do address EOL care - Author’s opinions: medical educators might have an underlying assumption that students can perform on their own on one of the most difficult tasks that physicians cant do themselves   Guidance and role modelling:   - From students: no one thought of asking a physician for help/advice when faced with difficulties with EOL care, students assumed residents were too busy - Author’s opinion: no one ventured into the possibility that medical educators themselves aren’t trained in this area - From literature: residents felt less competent in discussing EOL care as may feel it is in low priority.   Preparation for EOL care:   - From students: EOL care can only be learned through experience - From literature and author’s opinions agrees with students   Conclusion and recommendations:   - Both the current literature on education for end-of-life care and the medical students’ narratives agree that clinical experiences with dying patients are vital, role models are critical, medical educators should acknowledge the unspoken difficulties - Medical students should realise that preclinical EOL training is limited at the bedside | The formal curriculum does not train medical students adequately for their first clinical encounter of EOL care as medical educators failed to design effective means to provide it.  Although role modelling was critical in learning EOL care, no students nor looked to physicians for help. Both students and patients assume that physicians are too busy to discuss EOL care.  It is postulated that physicians themselves are inadequately trained in this area and hence leave medical students to tackle EOL care alone.  The only way to learn EOL care is through experience: understand the patient as a holistic human being and learning to view death postiviely. | NA | 15 |
| A. K. Henderson 2004 | The perfect hospice patient | A. K. Henderson recounts his experience as a 3^rd^ year medical student in hospice care while having very little experience in EOL care. She draws conclusions based on his reflection of this personal encounter with death | Not explicitly stated. A personal recount and reflection of death. | The author draws conclusions from two sources:   1. Lessons learnt from the attending physician: she has done everything she could have done, and the patient’s death was not her fault 2. Lessons from her reflection:  - Do not give false hope but encourage the will to live in the context of the illness - Good care is helping people approach death in a state of preparedness and peace - Be trained to recognise the last hours or days of life, to prepare yourselves and the patient’s family - We fail to be taught that death has the potential of being very special and positive if there is adequate preparation - Patients should die with dignity and the way in which they always wanted to - The highest quality of care can only be provided when providers are true to themselves by connecting with their innate ability to love and relate to other people | Medical students are ill prepared when facing EOL care as the formal curriculum does not prepare them adequately to manage the patient holistically.  Medical students learn how to deal with death and dying through experiences.  Medical students have the capacity to view death as a positive experience when one is prepared for it after learning from past experiences.  Advice from the faculty helps the student to reconcile their feelings of guilt when a death occurs.  Patient autonomy and respecting their wishes is part of good death – determine how he wants to die. | NA | NA |
| S. M. Shunkwiler, A. Broderick, R. B. Stansfield and M. Rosenbaum 2005 | Pilot of a hospice-based elective to learn comfort with dying patients in undergraduate medical education | The authors describe the effects of an innovative pilot program aimed at increasing students’ exposure to and understanding of issues faced by patients at the end of life | Quantitative and qualitative data was collected:  Quantitative:   1. Self-rating questionnaire: on emotions and attitudes about death and dying at three points in time: before training, after training, and after placement – ANOVA was used for analysis for 11 items 2. The “Attitudes About End-of-Life Scale” developed by Block and colleagues for use in a course for medical students at Harvard Medical School – a multiple regression model was used for analysis of 33 items 3. A Likert-type course evaluation questionnaire on barriers to participation and the usefulness of the components of an end-of-life course.   Qualitative data:   1. Tape recorded interview 2. Student journals   It is implied that thematic analysis was used to analyse the qualitative data | 30 students expressed interest in the study. Only 15 were recruited: 6 M1s, 5 M2s, 3 M3s, and 1 M4. Students went through hospice training and hospice placements during the course.  Quantitative data:   1. Self-rating questionnaire:    1. after placement, Participants rated themselves more comfortable with the physical manifestations of patients’ being at the end of life and less fearful of not knowing what to do or say around a dying person compared to before the placement    2. however, there was no statically significant difference between the responses of the participants who placed and those who did not place post training. 2. Attitudes about end of life:    1. after training, participants rated caring for dying patients as less depressing, and believed more strongly that practitioners were responsible for providing bereavement care to family members after a patient’s death. 3. Course evaluation:    1. Practical and scheduling difficulties (“exam/school demands” and “distance to assignment”) appear to have been more of a problem in joining the programme   Qualitative data:   1. Comfort with dying patients was increased 2. There was role ambiguity during the placement 3. Opportunity for reflection: being ready for death, confrontation of spiritual beliefs, death as a tedious long processes, death being best view away from the physician 4. Value of nonclinical aspects of dying and student autonomy: students could approach patients on their own to get to the patient as a person and not a disease 5. Student’s suggestions for the course: role playing, increased patient contact, more group meetings after patient contact to promote reflection, extension of the course to a year, more medically based for year 3 and 4s. | After going through hospice training and placement, medical students become more comfortable with death and become more knowledgeable with EOL care.  Most medical students enter the course with anxiety as to how to handle death.  Medical students view death as a positive item after their experience in the hospice.  Medical students knowledge the need to learn EOL care as part of medical training only after going through the hospice placement. | 7 | 14 |
| J. Rhodes-Kropf, S. S. Carmody, D. Seltzer, E. Redinbaugh, N. Gadmer, S. D. Block and R. M. Arnold 2005 | This is just too awful; I just can't believe I experienced that...: medical students' reactions to their "most memorable" patient death | The authors designed this study to assess the experiences of third-year medical students caring for patients who die. They wanted to understand students’ emotional reactions to their “most memorable” patient death, how students cope with their patients’ deaths, how much support students receive from the medical team in dealing with death, and the messages students receive from supervising physicians regarding death | Not explicitly stated.  Methods:   1. A semi structured interview about students’ education in end-of-life care during each of their third-year rotations and their satisfaction with this training. 2. a series of qualitative and quantitative questions about the “most memorable” patient death they had experienced during their third year. The quantitative questions air used ten point scales on 11 items.   Grounded theory was used to identity themes from the data and these were triangulated with quantitative data to ensure data validity. Quantitative descriptive techniques were used to describe certain themes from the interview data | 38 3^rd^ year medical students from two different schools completed the interview and questionnaire. Their demographics were roughly the same.   1. Characteristics of the “most memorable death”    1. Predominantly elderly    2. Most common cause was cancer (n=11)    3. Most were on IM rotation (n=19)    4. Students were only assigned to the patient’s care in 10 of the cases    5. Only 4 cases were the students very close to the patient 2. Themes from interview and questionnaire    1. students experienced strong emotional reactions to the patient deaths: when there was increased emotion, it resulted in a more disturbing death, new experience, finality of death, emotional experience were tied to how the patient reminded the patient of someone whom they are close to, raised questions about their competence    2. students used multiple strategies for coping with their emotional reactions to the death: talking to people, taking their mind off death, focusing on work and studies, praying, none involved the school or faculty    3. students perceived a lack of support from the medical team: in the 10 cases that death was discussed, 4 focused solely on the medical aspects, medical teams had insensitivity to patient’s death    4. death and emotions are viewed as negative aspects of medicine: inferred by the students due to the lack of discussion, patient’s death is equivalent to failure | A patient’s death can be memorable for a student even if the student did not know the patient well or for long.  Students viewed the medical system as ignoring their emotions and avoiding discussions of death, thus acculturating them to view emotions and death as negative aspects of medicine.  Most students had to rely on their own coping mechanisms with dealing with death as death was views as normality in the wards.  Without proper role modelling, the right balance of detachment and compassion may not be achieved in medical students  There is a viscous cycle of the formal curriculum being unable to meet the students needs as the faculty themselves aren’t trained well in handling death as a topic.  A unique opportunity to teach about death, emotions, and coping with stress was lost | 8 | 18 |
| N. Ratanawongsa, A. Teherani and K. E. Hauer 2005 | Third-year medical students' experiences with dying patients during the internal medicine clerkship: a qualitative study of the informal curriculum | The authors wanted to understand how third-year medical students, already trained with preclinical EOL curricula, experience death and dying in their first clinical clerkship in medicine. With focus on students’ experiences with death and dying during the core medicine rotation, students’ responses tot hese experiences, effect of prior EOL education affect these clinical experiences | A qualitative methodology was adopted. It was implied that thematic analysis was used to process the data.  Semi structured interviews were conducted. The interview included open-ended questions to elicit students’ experiences with dying patients, reactions to these experiences, coping strategies, and opinions of the value of prior preparation in EOL care. | Purposeful sampling of 32 students was done, targeted students that rotated through their first core IM clerkship. 28 were interviewed for 15-60mins.  Themes:   1. Relationship with patients: attachment to patients., Empathy for patients when comparing them to student’s own family members, advocacy motivated by a desire to help their patients in any way possible 2. Team interactions: acknowledgement – through explicit demonstrations was mostly not done, role modelling was the main mode of learning EOL care, lack of opportunities for active student participation resulted in a frustrated student 3. Student’s background: prior education in preclinical years did help to prepare students for EOL care but it had less impact than clinical experiences, those with personal experiences tended to be more comfortable working with dying patients during the clerkship, coping strategies included exercise, writing, music, therapy and prayer 4. Professional Identity: students learned how a professional expresses emotional response to care, transition – worried that their capacity to care for patients would diminish, self-efficacy – some teams viewed death as a negative outcome but even more medical teams viewed death positively | An experience with a patient’s death affects a student’s professional development.  The main mode of learning how to handle EOL care is through role modelling and experiential learning.  Students wished for the medical team to take more explicit actions to acknowledge a death.  There are notable cases of healthcare teams that taught students to view death positively and this resulted in an increased ability of the student to handle death. | NA | 19 |
| S. D. Block and J. A. Billings 2005 | Learning from the dying | The authors reflect on a personal reflection written by a medical student on his experience with a patient’s death. | Not explicitly stated. An expert’s commentary on a student’s reflection. | - Student’s tended to avoid the sadness, hopelessness, and helplessness they had associated with dying persons - it is replaced by a sense of the approachability of the dying, an interest in the medical, psychosocial, and spiritual aspects of “the case,” and a belief in the possibility of doing good work through such encounters - students learn to elicit and value the patient’s perspective - Students understand how they can be healers, even in the face of a terminal illness. | Experiencing a patient’s death can transform a student’s understanding and approach to death from something negative to a more positive and holistic outlook. | NA | NA |
| R. Fernandes, W. Shore, J. H. Muller and M. W. Rabow 2008 | What it's really like: the complex role of medical students in end-of-life care | The authors wanted to find out how medical students deconflict the following: hidden curriculum and dominant cultural milieu, training experiences differing from formal teaching and fluctuating roles and relationships. | Not explicitly stated. A qualitative analysis of tape tapes of a panel discussion about EOL care. The panel consisted of year 4 students and residents on EOL care rotations. Thematic analysis was done. | 70 2^nd^ year medical students attended this biannual panel.  Themes:   1. Defining professional identity: understand the “appropriateness” of expressing their identity 2. Conflicting expectations: students had to negotiate between advocacy for the patients and family and their alliance with the team 3. Limited medical experience: despite limited medical experience, students had were required to display true clinical skills | Although student have been theoretically trained on EOL care, there were some aspects of care that could not be transmitted through the curriculum.  Listening to experiences from role models seems to be able to fill in some of these gaps and legitimize student’s emotional reactions. | NA | 15 |
| W. G. Anderson, J. E. Williams, J. E. Bost and D. Barnard 2008 | Exposure to death is associated with positive attitudes and higher knowledge about end-of-life care in graduating medical students | The authors sought to empirically evaluate the association between exposure to death and medical students’ attitudes and knowledge about end-of-life care. They hypothesised that students who were exposed to death, either in their personal lives or during medical school, would have more positive attitudes and greater knowledge about end-of-life care | Not explicitly stated. A quantitative analysis using a survey for graduating classes that measured:   1. personal experience with death (loss of a close friend or family member) 2. whether they cared for dying patients or witnessed a patients’ death during their third-year clerkships 3. how many dying patients they cared for during their third year 4. how many deaths they witnessed during their third year 5. how many dying patients they helped care for over the course of medical school   This was done by a 4-point Likert scale with 8 statements, eventually this was converted to agree or disagree. There was also a knowledge-based questionnaire that consisted of 15 questions on true and false. Pearson X^2^ test was used for discrete variables.  There was another survey during their entrance to medical school to assess their interest in EOL care and the importance of caring for dying patients to them. | Class 2001-2006 had survey results on graduated students while entrance surveys were only available for 2005-2006. There were 380 graduating students with a response rate of 47%. Both entering and graduating surveys were available for 50 students.  Exposure to death:   1. 71% had an experience with death and 73% had cared for dying patients. 2. During their third-year clerkships, students cared for a median of 2 dying patients and witnessing a median of 1 death.   Attitudes towards EOL care:   1. students who had negative emotions at entry, emerged with positive views upon graduation 2. students with positive emotions retained those emotions   knowledge about EOL care:   1. mean results was 79% for graduating students   Relationship with exposure to death, attitudes and knowledge about EOL care:   1. Students who reported personal or professional experience with death were more likely to have positive attitudes about physicians’ responsibility and ability to care for dying patients and their families – dose effect between exposure to dying patients and attitudes and knowledge | Most students graduated with positive views on EOL care and about physicians’ responsibility and ability to help dying patients and their families, but reported negative emotional reactions to endof-life care.  Findings in this study contrasted medical literature – it could be because there was a compulsory palliative care programme that all students had to go through. | 8.5 | NA |
| E. Kelly and J. Nisker 2010 | Medical students' first clinical experiences of death | The authors wanted to construct a model of medical students responses to patient death. With focus on medical students’ ﬁrst experiences of the death of a patient in their care, particularly the interaction of reﬂection, emotion, pre-clinical education and the building of professional identity. | Thematic analysis by using grounded theory on a range of qualitative data telephone interview, focus group or through email with the same prompts.   1. ‘What happened the ﬁrst time you were involved in the care of a patient who died?’ 2. ‘How did the event make you feel?’ 3. ‘How did you cope with the event?’ 4. ‘How did the other health care professionals around you react to the event?’ 5. ‘Is there anything else important to you that hasn’t been asked?’ | Final-year medical students at the Schulich School of Medicine & Dentistry, University of Western Ontario were invited to participate in this research. 29 experienced a death during the course of their study and 20 agreed to be interviewed.  Axial results:   1. Preparation: medical student’s previous experience 2. Death event 3. Feelings 4. Role of clinical clerk 5. Differential factors between deaths 6. Closure 7. Relationships   Chronological model in 5 stages:   1. Preparation 2. Event itself: narrative of the event 3. Crisis: child death was perceived as a cause of greater emotional turmoil 4. Resolution 5. Lessons learned | There is strong tension between medical students’ charged emotional experiences and the detached professionalism that medical students perceive is expected of good doctors.  students who had the opportunity for discussion with supervisors, peers or friends were more willing to engage in the difﬁcult tension between emotion and professionalism.  Reﬂection and discussion about the death event are key to allowing medical students to integrate the apparent paradox of being professionally detached and emotionally involved with a patient’s death at the same time. | NA | 16 |
| C. Hsieh, C. A. Arenson, K. Eanes and R. D. Sifri 2010 | Reflections of medical students regarding the care of geriatric patients in the continuing care retirement community | The authors analyse the reflection papers written by 3^rd^ year medical students about their 1-day experience working in a continuing care retirement community (CCRC). | Not explicitly stated. A thematic analysis on opened ended reflection written by 3^rd^ year medical students about community health experience in the geriatric community. | In 5 years, 48 papers about the CCRC experience were collected which is 10% of all reflection papers:   1. Initial exposure to dementia: students found communicating with the patients to be difficult, realises that providing care to these group of patients is challenging 2. Confronting death and dying: initial fear and discomfort but eventually learn to accept that aging and death was a natural process 3. Diversity of care and services for the elderly: new appreciation of previously unknown types of care to the students 4. Cost of care for the elderly: students realise that financial and social status may affect the quality of care 5. Seniors can lead active lives if given the opportunity: elderly are seen in a different light – from sedentary lifestyles to active ones 6. Rewards of health care team-patient relationship: there is good relationships within the healthcare team and they see how treatment is balanced with comfort care at EOL | Student have improved attitudes and increased empathy on geriatric patients after this posting.  Students have their reconvinced notions of elderly care being depressing and sad altered to elderly care being rewarding and comforting at EOL. | NA | 10 |
| J. Frank 2010 | Refusal: deciding to pull the tube | Author reflects on his experience in the wards when a patient presents with a similar disease to her late grandfather. | A personal narrative reflection. | Initial thoughts:   - She was eager to do the right thing for Mr R - Student doctor who only sees black and white in moral dilemmas – pinned the fault on the family for not letting Mr R go peacefully   New insights:   - In retrospect, the family was not ready to accept that any treatments would be medically futile - Her decision to respect the elderly demented patient’s wish of rejecting the NG tube made her feel like a failure - Support from the attending physician regarding this decision made her shocked as she expected to be scolded - Experience shared from the attending allowed her to be more firm and at peace with her decision. | Medical students feel like failures if they are unable to carry out the orders from the doctors even if their patient does not want the treatment.  Medical students do not expect doctors to understand their emotions.  Medical students displaying emotions is not ideal.  When adequate support is given to student’s clinical decisions, they become more firm and have increased confidence | NA | NA |
| L. H. Jacoby, C. J. Beehler and J. A. Balint 2011 | The impact of a clinical rotation in hospice: medical students' perspectives | Authors aimed to describe the self-reported impact of a required one-week hospice rotation for third-year medical students. | A qualitative evaluation of reflective writing that aimed to enhance student’s self-awareness as well as to assess the effectiveness of their learning.  Not explicitly stated. A thematic analysis of the above. Themes chosen represented more than 30% of the students. | 104/137 3^rd^ year medical students completed the rotation and submitted essays.   1. Philosophy and benefits of hospice: leaning that hospice care is not giving up on the patient, learn to care for the patient holistically to increase their comfort 2. Professional impacts on students: better understanding of what to tell patients about hospice care 3. Interdisciplinary team approach: understanding how each member uniquely contributed to the team 4. Managing pain and suffering: multifaceted, beyond physical suffering, emotional and spiritual suffering 5. Facts about hospice care: new factual information about scope and type of services 6. Personal impact on students: student’s discomfort in communicating with dying patients was addressed, deeply moved by encounters with dying patients 7. Communicating with patients and families: new insights as to how to communicate | Exposing students to humanistic elements of EOL care helps to generate more competent doctors.  Observing EOL resulted in students being more comfortable with death and dying and in management of EOL patients.  Important sources of insights are the role models and patients. | NA | 11 |
| M. Baker, J. Wrubel and M. W. Rabow 2011 | Professional development and the informal curriculum in end-of-life care | Authors wanted to document the process of professional development training in EOLC during medical school. | A qualitative analysis of student narratives about EOLC training over 2-4^th^ year of school.  Semi-structured interviews were conducted annually.  Not explicitly stated. A thematic analysis was done. | 141 2^nd^ year students completed a survey on EOL care and 91 were willing to be interviewed. 10/91 was randomly chosen to be interviewed.   1. Professional development: learning could conflict with previously held value, value of experience with EOLC prior to medical school in helping solidify their professional values about EOLC 2. Curricular discordance: conduct of a medical professional through formal and informal curricula, and curricular messages were at times conflicting – some were encouraged to bottle up emotions while some were encouraged to share 3. Role models: were positive, negative or absent 4. Tightrope between trained vs human reactions: conflict between how medical professionals felt and dealt (distant) with EOLC with what was natural as a human being (acknowledgement) 5. Ethical dilemmas: balancing clinical care with learning, learning that sometimes there is no right outcome | EOLC experience during medical is essential for students to better understand how to care for dying patients.  Although tension and conflicts may arise – ethical, mismatched curriculum, students are able to negotiate and come to a compromise.  Role models are a big part of EOLC learning in medical students. | NA | 14 |
| M. V. Kavas and D. Oztuna 2011 | Thanatophobia in medical students: approach to death and dying patients attitude scale (ADDPAS) for undergraduate years in medicine | The authors seek to discuss the development of ADDPAS, a scale designed for medical students to evaluate the degree of thanatophobic attitude (defined as abstaining from death and dying patients and their relatives) using the Turkish language. | Not explicitly stated.  Data was collected and a tool was devised   1. Selection of model and working group: 436 volunteer medical students from the 4^th^ to 6^th^ years 2. Development of tool for data collection: comprehensive literature survey was done, alternative scales were found and combined into a new authentic scale after preview from professionals, final scale was shaped according to the views of two professors, 4-item Likert structure, first section has 5 questions about demographics and second section had 36 items in total to measure student’s ideals of death and dying 3. Exploratory factor analysis: items that had RMSEA <0.08 suggested a good fit and were kept 4. IRT model selection: produces latent trait person estimates which are independent of the distribution of the population, and item difficulty estimates which are independent of the ability of the person | 1. Exploratory factor analysis: 12 items were removed as they did not meet the dimension of “hardness in communicating with the dying and her relatives” nor “avoiding death and the dying”. 2. IRT model: a final 11 item sub scale | ADDPAS was created | NA | NA |
| J. H. Kearsley and E. A. Lobb 2013 | It is not a disease we treat, but a person: medical students' reflections on their first rotations to an oncology and palliative care unit | The authors wanted to analyze students’ reﬂections in order to determine the personal/professional impact of the rotation, to report on student responses to the new teaching curriculum, and to identify additional steps that could be taken to support students’ learning activities. | A thematic content analysis. | 54/90 reflections was selected after exposure to oncology and palliative medicine.   1. Listening to patient’s stories: communication with a dying patient was challenging, an effective way of establishing rapport 2. Communication: first used avoidance but afterwards formed a holistic picture of the patient after employing silence 3. Providing whole person care: patients are more than just the disease and psychosocial history is important 4. Increased confidence: in taking histories 5. Change in view: palliative care was initially regarded as a failure but they now understand that they need to move beyond cure and learn to comfort. | students perceived that they had undergone a positive personal transformation following their exposure to oncology and palliative care patients and their families.  Medical students reflective narratives were very important.  Students commented positively on communication skills improving. | NA | 10 |
| R. Spencer 2013 | A piece of my mind. Portrait of an artist | The author reflects on a recent death of a patient, | A personal narrative reflection framed as a letter to the patient who has passed. | Initial thoughts:   - Student was focused on medical aspect of the patient - Put distance in the relationship when the student sensed the patient was going south - Gratitude - Sense of profound loss and guilt   After reflection:   - Gratitude sounded like bravado - Human element into medicine – half of her is a doctor and the other half a human - Realised intimacy with the patient was an accidental result of her curiosity of the patient’s tumour   Current status:   - Thinks of the patient everyday - Visits the blog everyday | A patient’s death is a emotional experience (loss, guilt) that is long lasting.  Students start off wanting to understand the physical part of the patient, the psychosocial part only comes in much later after an intimate relationship is formed. | NA | NA |
| M. A. Mohd Slim 2013 | The superhero mythos: a medical student's experience of death | The author reflects on a recent death of a patient | A personal narrative reflection on experiencing death as a medical student. | When the patient crashed:   - Student was unsure of what to do during the chaos - Found himself unprepared for what was about to happen   After the death:   - Unsure how to process the sudden situation - Medical team did not address it   After reflection:   - It is easy to assume that medicine is about saving lives – due to society’s influence and being a bright eyed medical student - Sometimes a doctor does not do anything at all out of respect of the patient’s wishes or to allow the patient to leave the world with dignity | A death can result in medical students rethinking their definition of medicine and reframing it into one that cares instead of one that saves. | NA | NA |
| R. Jones and F. Finlay, 2014 | Medical students' experiences and perception of support following the death of a patient in the UK, and while overseas during their elective period | Given that students’ experience of death while overseas on elective periods has not previously been reported in the literature, the authors aimed to investigate medical students’ experiences and perception of support following a patient’s death, contrasting the experiences in the UK and while overseas during their elective period | A qualitative study was conducted. Convenience voluntary sampling was employed to select participants from a pool of final year medical students in one medical school. Data collection was done via anonymous online questionnaires. The results were analysed both quantitatively and qualitatively. | Only 131 of 220 questionnaires were returned, a response rate of 60%. Themes emerging from the qualitative responses included:   1. **‘Dealing with emotions’** – Students had mixed emotions following a patient’s death. In the UK, students reported feeling shocked, upset and sad. When overseas, many students were angry or frustrated, and many reported feelings of injustice. In the UK, 40% felt they had long enough for reflection before going back to work; On elective, only 16% felt they had enough time to reflect on a patient’s death. 2. **‘Global support networks’** – In both instances, the majority found talking to someone beneﬁcial. On elective, students looked globally for support turning to friends and family ﬁrst, rather than receiving support locally. Some students mentioned that they would have liked more general support. They suggested having an emergency contact number to reach a faculty member at any time. 3. On elective, only 14% (8/56) of students were involved in a debrief following a death compared with 37% (21/58) students in the UK.   **‘Best teaching methods’** – Only 13% (16/125) of medical students thought their course had prepared them sufﬁciently to deal with patient death. Of those who did feel prepared many said had gained this knowledge in ways outside of school. Prior to their elective period, students commented that they would beneﬁt from a lecture on ‘dealing with death’ and a lecture on ‘medicine in resource poor settings’. | 1. Students feel ill prepared for experiencing the death of a patient. Even though they may have ‘medical knowledge’ they are still lacking in emotional support and are often inadequately supported around the time of a patient’s death. 2. Medical schools need to introduce the theme of ‘death and dying’ at an early stage of the curriculum, and should make students aware of the possible experiences and emotions following the death of a patient and give them advice on whom to turn to for support while at home and abroad. | 6.5 | 11 |
| M. Vallurupalli, 2013 | Mourning on morning rounds | Reflective essay of a medical student centering on her experience at a palliative care rotation. |  | 1. **Detachment of the team from the death of a patient** – The team failed to acknowledge her passing, and in doing so we dehumanized her. Some silently suffered. 2. **Sense of failure** – Despite only being peripherally involved, the author felt a sense of failure and noted that the intern was second-guessing his decisions.   **Trainees suffered alone without guidance** from seniors and struggles for closure without time for reflection. | 1. Current training deemphasizes the ways in which illness and suffering affect others and trainees as they care for patients and experience loss. 2. This is unfortunate as their understanding of suffering is fundamental to the quality of the care that they give. 3. Implementing “death rounds”, where trainees had the opportunity to discuss the emotional impact of and insights gained from the deaths of their patients, could help support trainees emotionally, and allow them time for closure. These sessions can also develop skills that could help them care for critically ill patients in the future.   The author believes that we must find ways to memorialize the connection between patient and doctor in death as in life. | NA | NA |
| A. Harvey, 2013 | Preconceptions in palliative care: a medical student's perspective | Reflective essay after an eight-week summer elective split between the local hospital palliative care team and a local National Health Service hospice. |  | 1. **Challenged Preconceptions** – From viewing palliative care as synonymous with dying and pain control to viewing it as being able to offer much more than that involving multidisciplinary care beyond just pain control. 2. The author learnt the importance of emotional, spiritual, and cultural needs of the patient, aspects she says are often overlooked in other areas of medicine. 3. **Learnt how to break bad news by observing** – realize that even though bad news cannot be broken gently, it can be given sensibly and sensitively, with attention to the environment. 4. **Learnt the importance of caring for the patient’s family** – Communication with the patient’s family through every step of decision making was key in providing the best possible care for the patient. It also became apparent to me that the majority of patients felt an element of guilt for the burden they believed their illness had placed on the family. 5. Lack of entrenched hierarchies in palliative care – Author observed effective communication between health care professionals. | 1. Many preconceptions about palliative and hospice care exist that may be changed with an immersive attachment. 2. Greater emphasis must be placed on palliative care within educational strategies so that preconceptions that many students and patients hold regarding end-of-life care are challenged. With this, the author feels that the next generation of doctors will be even better equipped to deal with what can be a very challenging, sensitive, and rewarding area of medicine. | NA | NA |
| Kuczewski et al, 2014 | “I Will Never Let That Be OK Again”: Student Reﬂections on Competent Spiritual Care for Dying Patients | The authors aimed to examine medical students’ reﬂections on the spiritual care of a patient who has died so as to understand how students experienced this signiﬁcant event and how they or their teams addressed patients’ spiritual needs. | A qualitative study was performed. Data collection was done via reflective essay assignments of death of a patient. Students were randomized such that a random group of students wrote papers that were included. This prevented self-selection bias. Coding of the student essays consisted of an iterative, multistep process using a content analysis approach. The authors coded the essays for themes based on the competencies (developed by Puchalski and colleagues and reﬂected in the essay prompt). | 4 themes emerged from the 68 students’ responses of their care of a patient who had died:   1. **Communicating the prognosis of death** – Students did not always feel empowered to broach the topic of death if the team leaders had not. Essays frequently revealed that faculty and resident physicians are doing good work in telling patients and family members about a foreseeable death. 2. **Compassionate presence** – compassionate presence is not a single technique but a way of comporting oneself to signal warmth and care for the patient. 3. **Patient care** – referring patients to pastoral care, participating in shared prayer when invited, addressing feelings of guilt and recrimination, reaching across the fragmentation of the health care system, and tailoring treatment plans to meet the patient’s goals. Students identiﬁed systemic fragmentation of patient care as a barrier to meeting patient needs and as a facilitator of provider desensitization. 4. **Personal and professional development** – Students reported being aware that they were becoming desensitized to the human dimension of care, and particularly to dying patients and their families. Students wished to learn to contain their emotions to better serve their patients, and they articulated a commitment to addressing patient and family needs. Students characterized the suddenness of death or a patient’s rapid decline toward death as surprising, shocking, or stunning. Some reported having no avenue for ﬁnding closure. Students noted feeling great empathy for patients and families. Students noted multiple self-care techniques including reflection writing and prayer. Students often expressed a kind of pledge to take certain steps in the future such as always addressing the spiritual and palliative needs of the patient or family and making pastoral care referrals. | 1. This study provided insight into what knowledge, skills, and attitudes students found most relevant in meeting the spiritual needs of patients as well as insight into the personal and professional development of medical students and suggest that medical schools should support students’ formation. 2. Teaching institutions should seek to standardize team protocols to formally recognize and process patient deaths. 3. Educators must ﬁnd a way to support students in their wish to remain connected to and be present for dying patients and their families. 4. Educators must also ﬁnd ways to reinforce constructive behaviors. 5. Encouraging students to follow dying patients who have rotated off their service, allowing students to offer their pager number and physical presence to dying patients, and other like behaviors should be recognized as “best practices” among faculty. | NA | 11 |
| A. Hebblewhite, 2015 | A medical student's reflection on palliative care: Managing emotional connection with patients |  | Personal reflection by one medical student. | 1. Talking with Dr. M validated, for the author, that it is okay to become connected to patients so long as you are able to identify it and have a process by which to let it go. 2. The author’s discussion with Dr. M led her to further explore literature regarding how doctors engage with their patients emotionally and how this is viewed among medical professionals.   The author explores strategies that can be used to mitigate compassion fatigue and burnout and how to optimize interactions with patients to improve both the care that she offers and her own personal well-being – self-care, self-awareness, reflective writing, mindfulness meditation. | 1. The author mentions that with her conclusion of studies fast approaching, it is when she needs to develop the strategies to prevent compassion fatigue, burnout, and, ultimately, poor provision of care. 2. It is important to be able to ﬁnd colleagues to share these experiences. 3. By practicing self-care and self-awareness in the process of ongoing mindfulness meditation and reﬂective writing, the author has the opportunity to be invigorated and uplifted by the meaningful connections that she has with patients, reducing the chance of compassion fatigue and burnout and allowing her to provide the highest quality and most sensitive and engaged type of care. | NA | NA |
| Mott et al, 2014 | Medical Students as Hospice Volunteers: Reﬂections on an Early Experiential Training Program in End-of-Life Care Education | The authors aimed to evaluate the utility and impact of exposure to EOL care for ﬁrst year medical students (MS-1s) through a hospice volunteer experience. The speciﬁc aims were to determine whether such exposure would help increase students’ awareness of the physical, emotional, and spiritual dimensions of palliative care and whether this type of experience was appropriate for their level of medical training. | A qualitative study was performed. Students’ reﬂective essays on their experiences were analyzed using qualitative methodology and salient themes were extracted by two investigators independently and then collaboratively. | The following ﬁve themes were identiﬁed from students’ reﬂective essays:   1. Perceptions regarding hospice patients – Loss of independence and skills, need for human contact, normalcy of death, some students were surprised to ﬁnd how cognitively intact their patients remained until very close to death. 2. Feelings and reactions regarding self – apprehension and nervousness about the ﬁrst meeting with their hospice patient, unsure where to begin communication with their patients, what to expect, how to set the tone of the first visit. Generally, these feelings of discomfort were dispelled once their ﬁrst visit to the patient’s home was over. 3. Normalcy of EOL care at home – amazement at normalcy of dying at home. Maintaining this feeling of normalcy was noted to have a positive effect on patients and their families. 4. Impact of witnessing death and dying – Whirlpool of emotions, grief. Many students grew attached to their patients and felt helpless at being unable to change the circumstances. Triggered thoughts about their own mortality, the value of hospice volunteering, sacriﬁces of caregivers, and the courage and bravery of hospice patients. 5. Suggestions for improving EOL care education for medical students – desire for more training, a need to learn empathetic communication skills, ways to help both patients and caregivers, and knowledge of additional means of support for hospice patients beyond medications. | 1. Hospice volunteering during preclinical years may provide valuable experiential training for MS-1s in caring for seriously ill patients and their families by fostering personal reﬂection and empathic skills, thereby providing a foundation for future patient encounters during clinical training. 2. The results of this study imply that the PFF program offers a model for seamlessly integrating the ‘‘human’’ aspect with the ‘‘medical’’ science of caring for patients and may aid in transforming medical students into competent physicians over the course of their medical education. Therefore, medical schools are encouraged to consider adopting this model into their curricula. | 14 | NA |
| Cripe et al., 2017 | Medical Students' Professionalism Narratives Reveal That Experiences With Death, Dying, or Palliative Care Are More Positive Than Other Experiences During Their Internal Medicine Clerkship | The authors aimed to discover whether professional development of medical students is differentially affected by DDPC (death, dying, and palliative care) experiences. They hoped that by bridging this knowledge gap, development of educational strategies to optimize students’ preparedness for primary palliative care can be devised. | A qualitative study was conducted. Purposive sampling was employed to select anonymous narratives related to DDPC. Random selection of control narratives was done after stratifying by year of submission. The analysis followed immersion/crystallization procedures described by Borkan. | 547 narratives with professionalism related themes were analyzed, of which 391 (9.6%) were DDPC narratives and 156 were controls.   1. The majority (70.7%) of the DDPC narratives portrayed a positive experience. 2. The analysis revealed proportionately more DDPC narratives involved themes of (1) empathy, (2) honest disclosure, and (3) patient autonomy. In contrast, proportionately more control narratives contained themes of (1) nonjudgment, (2) equanimity, and (3) clinical teaching.   Many of the narratives concluded with an explicit acknowledgment of a lesson learned from the event. All the lessons learned were positive, that is, students were able to use even the negative experiences to demonstrate the importance of the preferred behaviour. | 1. There was a relative scarcity of DDPC narratives. A prospective observational study is needed to ascertain whether the low rate of DDPC narratives observed in the current study is attributable to minimal exposure to DDPC or whether it is due to the trainee’s focus on the other demands of the clerkship. 2. The data do not confirm the impression in the literature that DDPC experiences during the undergraduate clinical years are especially negative influences in the lives of third-year medical students. 3. Medical students’ professionalism narratives about DDPC are positive and provide evidence that students generally appreciate the examples of professionalism demonstrated by resident and attending physicians. 4. Advances in the discipline of palliative care and increased awareness of the importance of palliative care likely contributed to the positive narratives. Further research is necessary to determine the relative contributions. 5. The current results provide educators an opportunity to refine their understanding of the positive and negative behaviors that potentially influence students’ professional development. In addition, clinical educators should emphasize the relevance of core professionalism competencies to the wide range of the students’ clinical experiences including DDPC. | 10 | NA |
| Minor et al., 2015 | A Team Reacts to a Patient's Death | The authors (2 doctors and 1 medical student) seek to describe their experiences with a dying patient and to honour her life. | 3 narrative essays. | The findings from the medical student’s essay from the medical student:   1. Initially expected her patient’s death to be a distant event without much sadness but in reality, the experience brought her numerous negative emotions – feeling powerless, shock, anger, feeling of injustice. 2. The author avoided her feelings as an initial coping mechanism before facing them which resulted in a new resolve on how she wanted to practice medicine.   The author saw her patient as an inspiration to her and had gained new insight into how she should practice medicine – personably and holistically. | - The experience of interacting with and caring for a dying patient can have a powerful impact on a medical student’s conception of how medicine should be practiced. - The extent of this impact seemed to correlate with the extent of the loss felt by the student. | NA | NA |
| Smith-Han et al., 2016 | “That's not what you expect to do as a doctor, you know, you don't expect your patients to die.” Death as a learning experience for undergraduate medical students | The authors seek to contribute to our understanding of how medical students become doctors, and provide insights into the role a medical school may play in this development by exploring what students learn from the experiencing the death of a patient. The authors had the primary aim of exploring medical students professional and emotional development, with a supplementary aim to explore how medical students experienced the death of a patient during their clinical years attending a medical program. In particular, they were interested in any change in students’ perceptions during their experiences of a death of a patient. | A qualitative study was conducted. Purposive sampling was employed to select participants. Data collection was done via individual semi-structured interviews. Grounded theory analysis was used to analyse the data. The software program ATLAS.ti was used for the qualitative analysis. | 10 students were available to be interviewed throughout the clinical years of the program. Three main themes were derived:   1. **Students’ reactions to death and their means of coping** – Experiencing the death of a patient led to students feeling emotionally diminished, a decrease in empathy to cope with the emotional pain and seeking encouragement through the comfort of colleagues. 2. **Changing perceptions about the role of the doctor, the practice of medicine, and personal identity** – This involved a change in students’ perceptions from an heroic curing view of the doctor’s role to a role of caring, shaped their view of death as a part of life rather than something traumatic, and resulted in them perceiving a change in identity including dampening their emotions.   **Professional environment, roles and responsibilities** – Students began to experience the professional environment of the hospital by witnessing the ordinariness of death, understanding their role in formalizing the death of a patient, and beginning to feel responsible for patients. | 1. Encountering dying patients facilitates the necessary change of students’ perception about the role of the doctor and of practicing medicine from that of curing perspective to a one of caring or ‘healing’. 2. Staff support helps students cope. 3. The health professional’s very way of dealing with distressing events is different from the newly arrived medical student due to routinization of the abnormal. 4. Staff development targeting a working knowledge of the hidden curriculum, along with the role staff play in exercising this influence, is vital in order to facilitate translating the distressing experiences students face into worthwhile learning experiences. 5. Student learning about death needs to include learning about the social organization and working life of clinical settings, an area currently omitted from many medical education curricula.  - Curricula involving death and dying should be “integrated, rather than isolated in the curriculum”. | 18 | NA |
| Boland et al., 2016 | Medical students writing on death, dying and palliative care: a qualitative analysis of reflective essays | The authors aimed to contribute to our understanding of how undergraduate medical students learn about and reflect on death, dying and palliative care during and after clinical placements. The study has two objectives:   1. To explore how medical students, learn about and deal with death, dying and palliative care during a clinical placement.   To use the analysis of reflective essays to provide insights to improve medical education about end-of-life care and/ or palliative care. | A qualitative study of anonymised reflective essays was conducted. Purposive sampling was employed to select the reflective essays from a pool of essays written by third year medical students. The anonymised data were managed using QSR NVivo 10 software, and a systematic analysis, following the main principles of a grounded theory approach, was conducted in three distinct phases (as developed by Strauss and Corbin): (1) open coding; (2) axial coding and (3) selective coding. | 54 essays met the inclusion criteria; 22 students gave consent for participation in the study and their 24 essays were included (2 students had 2 included essays). 3 overarching themes were identified:   1. **Emotions** – Students described feeling anxious before the placement and were worried about how they would control their emotions during an encounter with a patient. For some, the emotions were related to triggering memories of bereavements or serious illness in a friend or relative. For others, the thought that what they witnessed during their placements could happen to them or someone close to them triggered emotions. 2. **Empathy** – Students emphasised trying to develop a balance between showing empathy and their emotional state. Consensus from these essays that time management and doctors’ empathy were associated with clinical competence which the students aspired to.   **Experiential and reflective learning** – Students learnt a lot from clinical encounters and watching doctors manage difficult situations, as well as from their refection during and after the experience. | 1. Students learn from seeing doctors in these situations especially as many will not have come across dying and death before. 2. When a student has been recently bereaved, it can add to emotional difficulties, but might also give the student insight and empathy that can only come with this experience. 3. Balance of emotions and using empathy and applying this in the clinical situation as professionalism, which students found difficult, are developed by experience of seeing tutors/teachers in practice and by refection. 4. Findings confirm previous work describing tensions between old and new professionalism (for instance, detachment vs empathy; patient-centred communication vs paternalism). 5. Students need to be comfortable discussing how they feel with the doctors they are working with to aid this experiential learning. This requires engagement from tutors. 6. It is vital that students’ opportunity to reflect is optimised by skills training and that reflective essays are used to enhance learning in a specific area. Providing feedback to students is key to developing the skills to use reflective discourse constructively. 7. There also needs to be promotion of experiential learning in palliative care and dying and death as core to the curriculum. | 16 | NA |
| Batley et al, 2017 | The effect of patient death on medical students in the emergency department | The authors aimed to assess students’ reactions to patient deaths in the ED compared to an inpatient setting, including aspects of death that generated the most powerful emotional responses, coping mechanisms, and whether or not counseling before the ED rotation or debriefing after a death would be deemed useful. Ultimately, understanding medical students’ experiences in the ED may allow for better interventions to help students cope with potentially traumatic experiences. | A qualitative study was conducted. Convenience sampling was employed to select the study participants. Data collection was carried out via semi-structured, in-depth interviews. Interview data were analyzed for themes. Thematic content analysis was used to examine the transcripts. Themes or patterns within the data were identified in an inductive or bottom up way. The qualitative data analysis was then based on grounded theory to identify recurrent themes and emergent patterns. | The sample consisted of 16 students, 10 females (62.5%) and 6 males (37.5%), with a mean age of 23.7. The following themes (in relation to ED and inpatient settings) emerged:   1. **Context of death** – students generally reported a ‘sudden’ and ‘unexpected’ death when talking about deaths in the ED (vs ‘expected’ in inpatient setting). Deaths that are unexpected and involve the young were described as more emotionally moving. Relating death to personal experiences, including thinking about death of relatives as well as thinking about death generally, increased students’ emotional reaction. 2. **Interaction with patient and family** – active or passive or minimal interaction. Greater interaction generally led to greater emotional investment. Interaction with patients in the ED, was described in many cases as minimal. This, in turn, led to a less pronounced emotional reaction. 3. **Assessment of medical team** – The team’s behavior was most commonly described as professional, organized, systematic, calm, efficient in both settings. Students reported no emotional reaction from the team and even a sense of detachment.   **Psychological consequences and coping mechanisms** – A wide range of emotional reactions was expressed in the inpatient setting and were of a different nature to in the ED. More students in the ED reported being unaffected and regarded death as part of the job. In the ED setting, not being involved in the care of the patient for a lengthy period of time improved their ability to detach and lessened the overall impact. Some students expressed guilt about feeling numb and detached and uncertainty about the proper reaction to have. Physical reactions to patient deaths were comparatively rare. With regards to the cognitive aspect, thinking about the death and the idea of death seemed to be a recurring theme for some students. For more traumatic cases, the imagery of the patient death was remembered vividly and flashbacks were experienced when similar cases appeared. Students commonly reported wanting to distance themselves and detach as a way to lessen the emotional impact. A minority opted to talk to colleagues, friends and family, and ‘try to be involved in other things’ as coping methods. A large number of students (7) cited the ability to regulate emotions as a learning experience as well as a coping mechanism. Becoming more empathetic and gaining experience on how to deal with the family were also considered valuable learning experiences. | 1. Higher levels of interaction and involvement with patients and their families correlated with a greater impact the experience had on the students. 2. Dealing with deaths in the ED may require or demand a different set of skills from the student. Dealing with death in the ED produces reactions of different nature vs in inpatient settings. Deaths in the ED triggered reactions relating more to the suddenness that is common in the ED. As such. Deaths in the ED are often more traumatic with gory imagery. 3. Witnessing the family’s reaction to the death even if no direct interaction took place made patient deaths in the ED more impactful. 4. Emotional reactions to death are influenced by culture. Individuals of non-western groups tend to view emotions as highly pertinent to beliefs, with emotions more readily causing belief change when compared to western cultures. 5. Given the interest by students, developing and testing debriefing techniques and sessions to support students and physicians in the ED setting may be a beneficial next step. | 18 | NA |
| Mutto, E. M., Cavazzoli, C., Ballbé, J. A., Tambone, V., Centeno, C., Villar, M. J., 2009 | Teaching dying patient care in three universities in Argentina, Spain, and Italy | The goals of the study were to establish similarities and differences between the three universities on:   1. Students’ attitude toward dying patients and training in EOL issues before engaging in a direct professional relationship with them   The contribution made over 6 years by a medical school to its students by comparing ﬁrst- and sixth-year students’ attitudes and wishes regarding care of terminally ill patients and their relationships with them. | A qualitative study was conducted. Convenience voluntary sampling was employed to select the study participants. Data collection was carried out using anonymous, multi-centered comparative semi-structured 24 question survey. For statistical analysis, the one-way analysis of variance (ANOVA) and the Newman-Keuls multiple comparisons test were used. Calculations were done using the SPSS 15 package (SPSS Inc., Chicago, IL). | 1. Most of the students have a positive attitude toward end-of life issues and want to receive more training. 2. The attitude of the surveyed students toward dying patients was highly positive. Most students stated that they would feel comfortable listening to a dying patient. Almost all the students expressed a high personal interest in assisting dying patients, death, and the end-of-life process. 3. In many cases students declared they felt unprepared to care for dying patients. Students felt that training should be taught as a topic within mandatory clinical–surgical subjects. 4. 98% of students considered that death and helping patients to have a good death should be included in their training. 5. 6^th^ year students did not consider the death of a patient as a failure for the physician, they also believed that the dying process may have a positive sense in the patient’s life and that the physician should devote time to discuss death with the patient and the family. 6. 30% of 3^rd^ year students wish to avoid affective involvement unlike 6^th^ years. 7. Most of the students expressed a wish to prepare themselves to offer better care and beneﬁt the terminally ill patients. A second group wanted to avoid affective involvement and a painful personal experience. A third group expressed a wish for personal enrichment and growth.   They also perceived that this issue received more attention in the humanistic rather than the clinical subjects. | 1. Attitudes toward dying patients were highly positive. 2. This could reﬂect a positive effect of personal contact with the patient and academic training in the students’ views. 3. Students’ attitudes revealed high interest and poor training in end-of-life issues. 4. Even when teaching emphasizes humanistic skills in the first 2 years, the clinical years fail to support and undermines these attitudes and practices. 5. Some attitudes improved from ﬁrst to sixth year while others remained unchanged, which indicates that there is a positive albeit insufﬁcient effect of present medical education. 6. Medical curricula should be improved to adequately address these issues. 7. In order to overcome this deﬁciency a signiﬁcant step would be to formally include the teaching of palliative care. It could also be useful to include this as part of accreditation standards for medical programs. | 8 | 7 |
| Schwartz, C. E., Clive, D. M., Mazor, K. M., Ma, Y., Reed, G., Clay, M., 2005 | Detecting Attitudinal Changes about Death and Dying as a Result of End-of-Life Care Curricula for Medical Undergraduates | The authors aim to find out:   1. Are these two attitudinal measures (Concept of a Good Death and Concerns about Dying) responsive to changes induced by two undergraduate EOL curricula? 2. Do these two curricula (a year-long Elective and a day-long Inter-Clerkship for medical undergraduates) have an additive effect (i.e., taking both yields a stronger attitudinal change than taking only one)?   Are there attitudinal and sociodemographic differences between students who took the year-long elective EOL course and those who did not? | A case control design (n = 100) and a one group pretest–posttest design (n = 98) using two measures [The Concept of a Good Death measure and The Concerns about Dying Instrument], each consisting of a fixed set of descriptive statements, were used to evaluate the two educational interventions.  T tests and Fisher’s exact tests (for continuous and categorical variables, respectively) and Linear regression analyses were used to evaluate the students’ scores. | 1. Elective participants reported less concern about working with dying patients at the end of the course and increased their valuation of clinical criteria in thinking about a “good death.” 2. There were trends suggesting decreased general concern about dying and increased valuation of closure, and an interaction suggesting a larger impact on those with higher precourse concern scores. 3. There were no differences between elective and nonelective participants at baseline. 4. The inter-clerkship increased students’ valuation of personal control aspects of death, and there was a trend in reducing concerns about working with dying patients. 5. No additive effect of taking both curricula. | 1. Results substantiate the effectiveness of both a year-long elective and a 1-day interclerkship in influencing students’ attitudes and beliefs about death and dying, and in increasing their reported level of comfort in working with seriously ill patients. 2. The longer curriculum seems to affect the value student placed on more psychosocial and existential concerns (i.e., closure), whereas the shorter curriculum appeared to affect the importance of patients’ control over bodily function. 3. Both measures were responsive to the relatively large effects this study would have been able to detect, and may be useful in future research to substantiate the effectiveness of EOL curricula in influencing attitudes and level of comfort with death and dying. | 8 | NA |
| Stepanyan, K. D., Weiss, T. E., Pessegueiro, A. M., Pietras, C. J., 2020 | Lessons From the Development and Implementation of a Palliative Care Elective for Fourth-Year Medical Students: A Pilot Study | Given the variable structure of education in palliative care and the common lack of outpatient exposure, the authors aimed to develop and implement a palliative care clinical elective for fourth-year medical students incorporating both inpatient and outpatient learning. | Data collection was via post-elective focus group discussions and Pre- and post-elective surveys assessing self-rated competency and attitudes towards caring for palliative care patients were used to evaluate students’ experiences.  Paired-sample 2 tailed t tests were run comparing pre- and postelective survey mean scores within each domain. | Post-elective,   1. Significant improvements in self-rated competency were seen in pain and symptom management, communication and advance care planning. 2. Survey results also showed improvement in attitudes toward caring for dying patients, with lower scores at the end of the elective suggesting reduced emotional distress. 3. Data revealed the most highly valued component to be direct observation and feedback during inpatient time. | 1. Findings reinforce the importance of prioritizing training palliative care faculty and team members in giving high-quality and timely feedback. 2. Future work should be targeted toward enhancing the quality and timeliness of feedback delivered by the palliative care interdisciplinary team. | 8.5 | 8 |
| Bertao, C., Kayashima, R., Braun, K. L., 2003 | Perceptions of a Required Hospice Experience: A Comparison of First- and Fourth-Year Medical Students | After receiving students’ comments about their experiences at the mandatory 12-week hospice unit, the authors sought to test the hypothesis that fourth year students were more appreciative than first-rear students of the program. | Data collection was done via a 32-item anonymous survey which tapped perceptions of usefulness of the unit, comfort with EOLC knowledge and skills, and suggestions for improvement. Data were analysed using SPSS. | 59 first years and 52 fourth years completed the survey.   1. Ninety percent of graduating students rated the hospice experience as personally valuable and 81% acceded its important for medical education, compared to 69% and 65% of first-year students, respectively. 2. Almost all felt that the hospice unit was a useful experience for them as future clinicians. 3. There were few differences in comfort with end-of-life skills between the two classes, e.g., almost 90% felt comfortable listening to patients; about 50% felt comfortable with their knowledge of grief/bereavement, symptom control, physical needs and psychosocial needs; and only 25% felt comfortable discussing a patient's terminal illness with the patient and family. 4. About half of each class felt comfortable recognizing, and coping with personal reactions to illness and death. 5. Only 30% to 40% of each class felt comfortable discussing advanced directives and DNR orders. 6. 50% of less in either class rated themselves comfortable with understanding the spiritual emotional, and psychosocial needs of the dying understanding the physical needs of dying patients; and understanding symptom and pain management. 7. Many learnt the importance of respecting patient autonomy, managing pain and symptoms, and considering spiritual and cultural values when caring for the dying. 8. The majority of students felt the unit should remain a first-year requirement, but wanted more opportunities to attend dying patients, work with physician role models, and reflect on their experience with death and its meaning. 9. Students wanted more involvement of physicians as palliative-care role models that students could shadow and confer with. 10. About a third wanted more formal training in palliative care from these experts. 11. About half wanted more contact with hospice patients and families.   Students wanted more opportunity to discuss and reflect on EOL issues. | 1. It is important that palliative care education he provided early in a student’s training. 2. Expanded experiences of graduating students likely influenced more of them to find the hospice unit personally valuable and important for their medical education. 3. Greater contact with dying patients, more opportunities for reflection, and increased involvement of physician role model experts is needed. This suggests a need for additional structured experiences in EOLC in the clinical years. | 9 | NA |
| Crawford, G. B., Zambrano, S. C., 2015 | Junior Doctors’ Views of How Their Undergraduate Clinical Electives in Palliative Care Influenced Their Current Practice of Medicine | The authors aimed to explore how junior doctors (doctors in postgraduate training) retrospectively perceived the influence of their undergraduate palliative care attachments (clinical electives) on their current medical practice | A qualitative study was conducted. Purposive sampling was employed to select the study participants. Data collection was carried out using one-on-one, semistructured interviews. Interview data were analyzed for themes. Analysis of the interviews, following Braun and Clarke’s approach to thematic analysis, was conducted to identify participants’ perceptions of the influences of palliative care attachments on their current clinical work. | 12 of the 14 junior doctors who had been invited responded and agreed to be interviewed. Sampling stopped at nine participants (six women and three men), following the principle of data saturation. Interview transcripts were analysed and two themes of how they thought their undergraduate palliative care attachments influenced their present practice were identified:   1. From apprehension to gaining a sense of control – Participants perceived that the learning experiences from the attachments provided them with a sense of confidence and control over their interactions with dying patients and families.  - Gaining confidence in their own abilities to deal with death and dying. - Learning about coping with death and dying. - Accepting death.  1. Gaining perspective on the practice of medicine –allowed them to identify aspects of palliative care that influenced them as medical practitioners beyond the palliative care context contributing to their professional growth.  - Incorporating aspects of palliative care into other areas of practice. - Becoming advocates of palliative care. - Strengthening the sense of purpose in medicine. | 1. Findings suggest that junior doctors trained earlier in palliative care have enhanced competencies of professionalism, patient-centered medicine, psychosocial and spiritual aspects of palliative care, communication, teamwork, and self-awareness that lasted post-graduation. 2. Learning experiences from the palliative care attachments seemed to ease the transition from medical student to junior doctor. 3. Learning a palliative approach can help them make a difference in treating dying patients, but also in general patient care. 4. Therefore, physicians trained in palliative care may be better prepared to contribute to a health care system that is person-centered, ethically conscientious, and personally fulfilling. | NA | 24 |
| Gillett, K., O'Neill, B., Bloomfield, J. G., 2016 | Factors influencing the development of end-of-life communication skills: A focus group study of nursing and medical students | The authors aimed to explore factors influencing the development of end-of-life communication skills by nursing and medical students especially since many doctors and nurses find this a challenging area of practice despite it being a core part of nursing and medical education. | A qualitative study was conducted. Convenient voluntary sampling was employed to select the study participants from a population of second year nursing students and fourth year medical students. Data collection was done via five single discipline (2 with nursing students and 3 with medical students) focus groups was conducted. Analysis of the interviews, following Braun and Clarke’s approach to thematic analysis, was conducted. | Second year undergraduate nursing (n=9 across 2 focus groups) and fourth year undergraduate medical students (n=10 across 3 focus groups) participated in the study. Two main themes emerged:   1. Intrinsic factors influencing EOL communication.  - Barriers and facilitators to EOL communication that originated from students themselves. - Intrinsic facilitators included self-confidence, previous experience and relationships with patients. - Intrinsic barriers included not knowing what to say, dealing with emotional responses, wasting patients' time, and concerns about their own ability to cope with distressing experiences.  1. Extrinsic factors influence EOL communication.  - Extrinsic facilitators included theoretical classroom input, patients who were willing to talk, and role models.   Extrinsic barriers to communication included gatekeeping by qualified staff and lack of opportunity to make sense of experiences through discussion. | 1. In addition to clinical placements, formal opportunities for reflective discussion are necessary to facilitate the development of students' confidence and skills in end-of-life communication. 2. Students’ motivation to interact with dying patients might increase if EOL communication was included as a specific clinical learning outcome and therefor an unmistakably legitimate part of their learning. 3. Mentors and supervisors may require training to enable them to facilitate students to develop end-of-life communication skills and effective facilitation of communication with EOL patients. |  | 17 |
| Goldberg, G. R., Gliatto, P., Karani, R., 2011 | Effect of a 1-Week Clinical Rotation in Palliative Medicine on Medical School Graduates’ Knowledge of and Preparedness in Caring for Seriously Ill Patients | The objective of this study was to assess the effect of the addition of a required clinical rotation in palliative medicine on graduating medical students’ self-rated knowledge and skill level in caring for seriously ill patients, as well as their performance on a knowledge examination. It was hypothesized that the addition of the clinical exposure would result in higher self-rated knowledge and skills, higher ratings of the quality and quantity of their education in palliative care, and better performance on a knowledge examination of palliative medicine than for a historical control cohort of students who received didactic sessions on palliative care topics without clinical experiences. | A historical control trial was conducted. Voluntary sampling was employed to select the study participants out from the two classes. Data collection was carried out using anonymous online survey designed to assess experiences and preparedness in caring for seriously ill patients. Paired t-tests were used to compare scores on the knowledge examination for the classes of 2007 and 2008. Chisquare tests (or Fisher exact method for items in which data was very unequally distributed) were used to compare the online survey responses and the AAMC graduation questionnaire data from the two classes. Themes and categories were derived inductively from all qualitative comments using grounded theory and a constant comparative method of analysis. | Fifty-eight (55%) students in the class of 2007 and 59 (51%) in the class of 2008 completed the online anonymous survey. Ninety-four percent of students in the class of 2007 and 100% of students in the class of 2008 responded to the AAMC graduation questionnaire.   1. Students from MS08 rated their skill level in several areas of pain management and communication more favorably than did students from MS07. 2. Mean scores on the knowledge portion of the survey were not signiﬁcantly different between the two classes. 3. Students from 2008 felt the categories of useful skills learned during the rotation are communication (n=588), symptom management (n=547), and other (n=522). The three most frequent subcategories under communication were family meetings, breaking bad news, and end-of-life discussions. The symptom management category was further subdivided into pain, non-pain symptoms, and other skills.   Students from 2008 also responded to the open-ended question ‘‘How have you used these skills in your clinical practice?’’ Responses fell into three broad categories: communication (n=517), pain (n=517), and applicability of palliative care to other settings (n=516). | 1. Clinical exposure to hospice rotation has been demonstrated to result in an improvement in palliative medicine knowledge at the time of completion of the rotation but that did not last to make a difference in scores just before graduation. 2. Graduating medical students who had a 1-week clinical rotation in palliative medicine had higher self-assessed skills in pain management and communication than students who received no clinical exposure. 3. The study suggests that students felt that they could apply what they learned on palliative care to a wide range of settings and scenarios. 4. Hidden curriculum in the rotation inculcated skills in affective components of care, which included responses such as compassion, patience, and empathy, were not a part of the formal course curriculum. 5. A brief clinical experience in palliative care, to supplement formal didactics in teaching these skills, should be considered for integration into the curriculum at all medical schools. | 10.5 | 13 |
| Kriesen, U., Altiner, A., Müller-Hilke, B.,2018 | Perception of bedside teaching within the palliative care setting-views from patients, students and staff members | Given the increasing demand on end of life education in medical schools and the possible use of bedside teaching, the authors aimed to:   1. Analyze the perception of bedside teaching on a palliative care ward from the perspectives of students, staff members and patients; and   Define the prerequisites for an efficient and patient-centered bedside teaching on palliative care wards. | A qualitative study was conducted. Convenient voluntary sampling was employed to select the study participants from a population of students who enrolled in the elective course “Intensive Practical Training Palliative care”. Data collection was done via self-developed questionnaire before and after the course and semi structured interviews at a later stage. Data was later analysed according to the thematic framework approach.  Note: Members of the palliative care team were also invited to participate in this study | A group of 21 students in their clinical years, 20 patients and 19 members of the palliative care team participated in this study. Qualitative analysis of the data following the framework approach revealed five core concepts related to bedside teaching on the palliative care ward.   1. Best possible end-of-life education for students — students enjoyed being around real patients, their perceived learning input increased due to reality. 2. Benefit of bedside teaching for participants — Patients mostly wanted to help, students enjoyed the benefit of reality. 3. Gain in knowledge, skills, and competencies — knowledge, practical skills like communication with the terminally ill and dying patients. 4. Disturbances to patients and team.   Reflections on death and dying — Students participating in our seminar did not mention fear or grief to be a major issue after experiencing palliative care education at the bedside. | 1. The cohort of students confirmed the benefit of direct contact to patients in palliative care situations and thus support previous recommendations for thoughtful, integrative and interdisciplinary curriculum changes in end-of-life education as psychological and emotional experiences cannot be taught in the classroom. 2. Bedside teaching within the palliative care setting classified a valuable tool for students especially in learning specialized palliative care skills. 3. Subsequent to the bedside teaching on a palliative care ward, students should be given the opportunity to reflect on what they experienced. 4. Students mostly felt comfortable with palliative care patients and did not request professional help for coping with experienced aspects of dying and death. 5. Students’ experiences with dying patients are considered to induce a long-term change of attitudes. 6. However, in order to protect the critically ill, students need structured guidance and strict behavioral instructions for presence at the bed side. 7. While some of the patients suffered from the renewed confrontation with their own unfavorable fate, none of the students reported any distress from the contact with dying patients. | NA | 10 |
